# Transcriptome analysis of *Pseudomonas syringae* pv. tomato DAPP-PG 215 in response to silver nanoparticles exposure

**DOI:** 10.3389/fmicb.2025.1714857

**Published:** 2026-01-06

**Authors:** Benedetta Orfei, Joël F. Pothier, Luca Scotti, Antonio Aceto, Chiaraluce Moretti, Roberto Buonaurio, Theo H. M. Smits

**Affiliations:** 1 Department of Agricultural, Food and Environmental Sciences, University of Perugia, Perugia, Italy; 2 Environmental Genomics and Systems Biology Research Group, Institute of Natural Resource Sciences (IUNR), Zurich University of Applied Sciences (ZHAW), Wädenswil, Switzerland; 3 IIS “Alessandrini-Marino”, Teramo, Italy; 4 Retired, Teramo, Italy

**Keywords:** heavy metal resistance, RNA-seq, nanoparticle, antibacterial mechanism, biocontrol

## Abstract

Silver nanoparticles (AgNPs) are one of the most promising options for the control of bacterial pathogens. The effectiveness of AgNPs has been proven by several recent research studies in which toxicity against a broad range of human and plant pathogenic bacteria was observed, even when used at low doses compared to other conventional bactericides. Nevertheless, the antibacterial mechanism of AgNPs is yet to be completely understood. This study investigated the first response of *Pseudomonas syringae* pv. tomato strain DAPP-PG 215, the causative agent of bacterial speck disease of tomato, to AgNPs. Gene expression of *P. syringae* pv. tomato in response to AgNPs was observed after 10 and 30 min of exposure. A total of 78 and 66 genes were identified as differentially expressed, respectively. Gene ontology and KEGG pathway enrichment analysis revealed a high representation of genes related to stress resistance, energy metabolism, and metal ion binding proteins, with different transporters involved. Validation of four selected genes by quantitative RT-PCR analysis, confirmed these observations. These results not only supported initial findings on the antimicrobial action of AgNPs but also suggested a significant impact of different heavy metal and toxin transporters in the silver detoxification mechanism of bacteria.

## 1 Introduction

Nanoparticles are materials which size ranges between 1 and 100 nm (Nagarajan, 2008). Due to their nanometric size, they have a high surface area-to-volume ratio that confers them an enhanced catalytic activity and a peculiar set of chemical and physical properties, which may also differ from those of their bulk counterparts (Xie et al., 2020). For these unique characteristics, dependent on the size of the nanomaterial, nanoparticles are driving significant interest in research for their application in biomedicine, pharmaceutics, agriculture, food safety, cosmetics and environmental health (Stark et al., 2015; Harish et al., 2022). According to their composition, nanoparticles can be classified in organic and inorganic (Ealia and Saravanakumar, 2017). Among the second ones, metal and metal oxide nanoparticles have received increasing attention in therapeutics, where they are used as antimicrobial agents (both directly or as carriers), and for diagnostics purposes (Sánchez-López et al., 2020; Shabatina et al., 2022).

Silver nanoparticles (AgNPs) in particular, have proven to be promising in controlling human and plant pathogens due to their marked antimicrobial properties (Beyene et al., 2017; Xu et al., 2020; Dawadi et al., 2021) exerted against a large number of pathogenic bacteria and fungi. The toxic activity of AgNPs against bacteria has been exhibited in terms of growth, motility and reduction of the capability to form biofilms (Franci et al., 2015; Abalkhil et al., 2017), while against fungi, not only growth, but also spore formation and germination were affected (Ogar et al., 2015; Matras et al., 2022). AgNPs were capable of controlling several antibiotic-resistant human pathogens such as *Acinetobacter baumannii*, *Pseudomonas aeruginosa*, and *Staphylococcus aureus* (Mirzajani et al., 2011; Salomoni et al., 2017; Liao et al., 2019; Hetta et al., 2021). In agriculture, AgNPs can be used not only as a nanopesticide, but also as a nanofertilizer (Raliya et al., 2018; Khan et al., 2023), promoting plant growth and nutrient uptake. Moreover, when considering their antimicrobial activity, it was reported that, when used to treat susceptible plants previously or subsequently inoculated with pathogenic bacteria, they were able to reduce disease severity both directly, inhibiting *in planta* growth of the pathogen, and indirectly, by inducing the plant defensive response (Mishra et al., 2017; Kumari et al., 2020).

Although the natural antimicrobial properties exhibited by silver are well known for centuries, the exact mechanism by which this activity is exhibited is still under discussion (Mijnendonckx et al., 2013). Overall studies have suggested that the ionic form silver (Ag^+^) can alter interactions with sulfhydryl groups present on the pathogen membrane, preventing hydrogen ions from binding. This triggers the inhibition of cellular respiration and electron transfer, leading to an alteration of membrane potential, loss of membrane integrity, cell lysis and cell death (Russell and Hugo, 1994; Barras et al., 2018).

However, at the cytoplasmatic level, different mechanisms were involved following exposure to Ag^+^ ions. Indeed, following membrane damage, Ag^+^ ions can enter the interior of the cell and cause suppression of DNA replication, denaturation of ribosomes, and an increase of reactive oxygen species (ROS) production (Park et al., 2006; Yin et al., 2020). Consequently, the antimicrobial action of silver was imputed to multiple events (Vishwanath et al., 2022).

Regarding AgNPs, the mode of action involved the same events that underlie the antimicrobial properties of silver in bulk form, but the entire mechanism was regulated by factors such as the shape and size of the nanoparticles (Tang and Zheng, 2018). In fact, smaller nanoparticles can release more Ag^+^ ions, and can be more easily adsorbed within the cell, while shape can affect the aggregation state of the nanoparticles at the membrane level (Xiu et al., 2012). An important aspect that must be also taken into consideration is the bacterial response to exposure with AgNPs (Maillard and Hartemann, 2012). Resistance to silver in bacteria has been associated with the presence of energy-dependent efflux transporters, for which the gene is often located on plasmids. In the specific case of silver, the possibility that this metal may be accumulated on the cell wall has also been reported (Gupta et al., 1999, 2001; Silver, 1996; Silver et al., 2006). However, recent research suggested that the Gram-negative bacteria *Escherichia coli* and *P. aeruginosa* can develop a resistance mechanism and overcoming subinhibitory silver concentrations due to a consequent increased production of the bacterial flagellum protein flagellin, which resulted in the aggregation of AgNPs and suppression of their antibacterial effect (Panáček et al., 2018; Stabryla et al., 2021).

In this study, we investigated the *in vitro* effect of a subinhibitory dose of very small silver nanoparticles (AgNPs), defined as silver ultra-nanoclusters (Argirium-SUNCs), on the pathogenic bacterium *Pseudomonas syringae* pv. tomato, the causative agent of bacterial speck of tomato, at the transcriptomic level. Through RNA sequencing (RNA-Seq) and subsequent bioinformatic analysis, the differentially expressed genes of *Pseudomonas syringae* pv. tomato strain DAPP-PG 215 were examined. Validation through quantitative PCR (qPCR) confirmed the expression patterns observed by RNA-Seq.

## 2 Materials and methods

### 2.1 Bacterial strain and growth conditions

For this study, *Pseudomonas syringae* pv. tomato strain DAPP-PG 215 (Buonaurio et al., 1996; Orfei et al., 2023b) was selected. The strain was obtained from the collection of phytopathogenic bacteria of the Plant Protection Section of the Department of Agricultural, Food and Environmental Sciences, University of Perugia (Italy), where it was stored in vials containing 15% glycerol at−80°C. Revived on Nutrient Agar medium (NA; Oxoid, UK) at 27 ± 2°C for 48 h, strain purity was verified before usage by plating a single colony on plates containing NA enriched with 50 g L^–1^ of sucrose (Lelliott and Stead, 1987).

### 2.2 Response of *Pseudomonas syringae* pv. tomato to challenge with silver ultra nanoclusters

To study the first early response of *P. syringae* pv. tomato strain DAPP-PG 215 to silver ultra nanoclusters (Argirium-SUNCs), its gene expression was evaluated at different time points following exposure to a sublethal concentration of the silver nanoclusters. Argirium-SUNCs were synthesized by using a registered electrochemical protocol (EP-18181873.3), in ultra-pure water and without stabilizing agents, as reported in previous studies (Scotti et al., 2017; Pompilio et al., 2018; Grande et al., 2020; Molina-Hernandez et al., 2021; Borgolte et al., 2022). The sublethal concentration was chosen in accordance with the results obtained in a previous *in vitro* experiment with Argirium-SUNCs on the same bacterial pathogen (Orfei et al., 2023a), where a concentration of 0.125 parts per million (ppm), which is one fourth of the minimum inhibitory concentration (MIC), induced a reduction in bacterial growth of just under 50%. The following criteria were used for this transcriptomic study: three biological replicates per time point, three short-term sampling intervals (*T*_0_, *T*_10_, and *T*_30_, corresponding to 0, 10, and 30 min of exposure to Argirium-SUNCs), and two treatment groups for comparison (Argirium-SUNCs-treated and ultra-pure water-treated controls). This design resulted in a total of 18 samples for RNA extraction.

After verifying its purity by plate observation, a single colony of *P. syringae* pv. tomato DAPP-PG 215 was inoculated in KB (King et al., 1954) liquid medium and grown overnight at 27°C, providing continuous shaking at 200 revolutions per minute (rpm). The overnight starter culture was diluted 1:100 to inoculate 40 mL of fresh KB liquid medium, in a 250 mL autoclaved Erlenmeyer flask. The flask was cotton-plugged, corked with aluminum foil and incubated under the same conditions until the absorbance (*OD*_600_) of the culture reached 0.5 [equal to a bacterial population of 5 × 10^8^ colony-forming units per milliliter (cfu mL^–1^]. For RNA extraction, nine 100 mL autoclaved flasks, assigned as control, were filled with 4 mL of fresh KB liquid medium, 1 mL of the *P. syringae* pv. tomato 5 × 10^8^ cfu mL^–1^ bacterial culture, and 5 mL of ultrapure sterile water. Additionally, nine further flasks, assigned as Argirium-SUNCs-treatment, were filled with 4 mL of fresh KB liquid medium, 1 mL of the *P. syringae* pv. tomato 5 × 10^8^ cfu mL^–1^ bacterial culture and 5 mL of a 0.125 ppm Argirium-SUNCs solution. These subcultures were incubated at 27°C with continuous shaking at 200 rpm. Three flasks for control and three flasks for Argirium-SUNCs treatment were removed from the incubator for cell harvesting after a few seconds (*T*_0_), and the same practice was repeated after 10 min (*T*_10_) and 30 min (*T*_30_). The cells were harvested by transferring the volume of each flask into a 15-mL tube and centrifuging each sample at 4,000 × *g* for 15 min at 4°C. After centrifugation, the supernatant of each sample was removed, and the tubes were immediately dipped into liquid nitrogen, for further storage at−80°C.

### 2.3 RNA isolation and sequencing

RNA was extracted from the frozen cell pellets with the RNeasy^®^ Plus Micro Kit (Qiagen Europe) according to the manufacturer instructions. DNA contamination was removed by the columns provided with the kit. After confirming RNA integrity and purity via electrophoresis and measurement with a NanoDrop spectrophotometer, all RNA samples were prepared for shipment on dry ice and sent to Novogene Europe (Cambridge, England), where RNA quality control, library preparation, sequencing, and data quality control were conducted. Briefly, after receiving the samples, the RNA quality was assessed using NanoDrop, agarose gel electrophoresis, and an Agilent 5,400 Fragment Analyzer. Then, after ensuring rRNA removal, a directional mRNA library was prepared and tested for its quality. Finally, the Illumina NovaSeq 6,000 Sequencing System was used to perform a paired-end 150 bp sequencing of the samples. All data resulting from the sequencing were submitted for final quality control.

### 2.4 Bioinformatic analysis

Fastq data obtained from Novogene Europe, once assessed for quality with FastQC v.0.11.9 (Andrews, 2010), were submitted to Bowtie2 v.2.4.5 (Langmead and Salzberg, 2012) for the alignment with the *P. syringae* pv. tomato DAPP-PG 215 reference genome (EMBL assembly accession number GCA_949769235). The SAM files of the Bowtie2 alignment were converted into BAM files and sorted with SAMtools v.1.15 (Li et al., 2009). To assess the overall similarity of samples, principal component analysis (PCA) was performed on raw read counts obtained through featureCounts v.2.0.1 (Liao et al., 2014) using the rlog transformation implemented in DESeq2 v.1.32.0 (Love et al., 2014). Following that, Cufflinks v.2.2.1 (Trapnell et al., 2010) was used to assemble the individual transcripts that had been aligned to the genome. Then, following the Cufflinks workflow (Trapnell et al., 2012), these assemblies were submitted to Cuffmerge v.2.2.1 to merge each sample according to the biological replicates. The merged GTF file was then provided to Cuffdiff v.2.2.1 along with the alignment files produced from Bowtie2. Cuffdiff returned as output statistically significant changes in gene expression for each condition (Treatment × Time-point) based on an adjusted *p*-value (*q*-value) < 0.05. Cuffdiff output data were used for a further analysis conducted in R v.4.2.0 with the package CummeRbund v.2.0.0 (Goff et al., 2014), which allowed the visualization of the results generated by Cuffdiff. Differentially expressed genes (DEGs) were identified using a Log_2_-Fold Change (LFC) ≥ 1.5 and an adjusted *p*-value (*q*-value) < 0.05 cutoff. For functional annotations, DEGs sequences were subjected to online BLAST (Altschul et al., 1990) (performed on 15/05/2022) for similarity search against UniProtKB reference proteomes and Swiss-Prot databases (*E*-value ≤ 10^–3^). Enrichment analyses of gene ontology (GO) and local network cluster (STRING) were performed using the software ShinyGO v.0.82 (Ge et al., 2020), while enrichment of Kyoto Encyclopedia of Genes and Genomes (KEGG) pathways was obtained from the KEGG Orthology-Based Annotation System intelligent version (KOBAS-i) tool (version 3.0; Bu et al., 2021). Both GO, local network cluster and KEGG pathways significantly influenced by the different gene expression were obtained selecting the “*Pseudomonas syringae* pv. tomato DC 3,000” database as reference and false discovery rate (FDR) < 0.05. Raw reads for all samples of this study are available at NCBI Gene Expression Omnibus (GEO) database (accession number: GSE308472).

### 2.5 RNA-Seq gene expression using RT-qPCR validation

Four differentially expressed genes (DEGs), identified at both *T*_10_ and *T*_30_ sampling points (Table 1), were selected for qRT-PCR validation of the RNA-Seq results. Among these DEGs, three were upregulated and one downregulated. The gene names, locus tags, primer sequences, and annealing temperatures for these genes, along with the reference housekeeping genes used for normalization, are provided in Supplementary Table 2. The primers were designed using Primer 3Web v. 4.1.0 software, confirmed using online BLASTn analysis at the National Center for Biotechnology Information (accessed on 18/03/2024)^1^ and custom-synthesized by Sigma-Aldrich, United States. Synthesis of cDNA was performed using the iScript cDNA Synthesis Kit (Bio-Rad Laboratories Inc., Hercules, CA, United States) according to the manufacturer’s instructions. Reverse transcription quantitative PCR (RT-qPCR) was performed on a CFX96 system (Bio-Rad, Hercules, California, United States), employing a 96-well plate with 20 μL of PCR reaction per well, containing 50 ng of cDNA as a template, 500 nM of each specific forward and reverse primer and 10 μL of SsoFast EvaGreen Supermix (Bio-Rad Laboratories Inc., Hercules, CA, United States). The amplification steps were 98°C for 30 s, followed by 40 cycles of denaturation at 98°C for 5 s and annealing for 5 s, and finally 65°C with a 0.5°C increase every 5 s up to 95°C, for fluorescence measurement. All biological samples were run in triplicate for each gene tested, and a sample without cDNA was added as negative control. Target gene relative expression levels were calculated comparing their threshold cycles (*Cq*) to the expression of the two references genes according to the Pfaffl (2001) method with modifications by Vandesompele et al. (2002). ANOVA and Tukey’s multiple comparison test were used to analyze the differences and estimate the level of significance between untreated control and Argirium-SUNCs-treated samples. Finally, the RNA-Seq results for the four DEGs and their relative expression data obtained via RT-qPCR and transformed into LFC were compared using the Pearson correlation coefficient for both time points (10 and 30 min).

**TABLE 1:** List of shared differentially expressed genes (DEGs) in *Pseudomonas syringae* pv. tomato Strain DAPP-PG 215 from samples exposed to Argirium-SUNCs for 10 min (*T*_10_) and 30 min (*T*_30_).

Locus tag	Annotation	Log_2_ fold change *T*_10_	Log_2_ fold change *T*_30_
DAPPPG215_26360*	Cadmium-translocating P-type ATPase	3.2943	3.9187
DAPPPG215_01280*	Efflux RND transporter permease subunit	3.0028	3.1790
DAPPPG215_01380	Arsenate reductase (EC 1.20.4.1)	2.0405	3.2679
DAPPPG215_06925	Type 3 secretion system secretin (T3SS secretin)	1.9090	2.5467
DAPPPG215_03455	Bcr/CflA family efflux transporter	2.1014	2.2348
DAPPPG215_19955	Protease HtpX (EC 3.4.24.-) (Heat shock protein HtpX)	2.5445	2.1289
DAPPPG215_01370	Urease accessory protein UreG	1.5086	2.1108
DAPPPG215_26355	Formimidoylglutamase, putative	2.0408	2.1056
DAPPPG215_25700	ATP-dependent protease subunit HslV (EC 3.4.25.2)	1.9571	1.9967
DAPPPG215_14185	Regulatory protein, putative	1.7636	1.9725
DAPPPG215_08170	ferredoxin–NADP(+) reductase (EC 1.18.1.2)	1.9324	1.9125
DAPPPG215_23735	Periplasmic ligand-binding sensor protein	1.8094	1.8668
DAPPPG215_24425	Curved DNA-binding protein	1.5138	1.8560
DAPPPG215_01365*	Fructose-1,6-bisphosphate aldolase (FBP aldolase) (EC 4.1.2.13)	1.6501	1.7911
DAPPPG215_27315	Phosphatidate cytidylyltransferase (EC 2.7.7.41)	2.0153	1.7547
DAPPPG215_14675	Sulfite reductase	1.7758	1.7485
DAPPPG215_22485	Cytoplasmic membrane family protein	1.6009	1.6800
DAPPPG215_22910	Hydrolase, carbon-nitrogen family	1.5657	1.5163
DAPPPG215_10650*	Conserved domain protein	−1.7671	−1.8287
DAPPPG215_00040	FAD/NAD(P)-binding domain-containing protein	−2.2969	−2.2272
DAPPPG215_00035	Metallo-beta-lactamase superfamily protein	−2.5607	−3.0645

Genes used in RNA-Seq validation are marked with an asterisk (*).

## 3 Results

### 3.1 Transcriptome analysis

**FIGURE 1:**
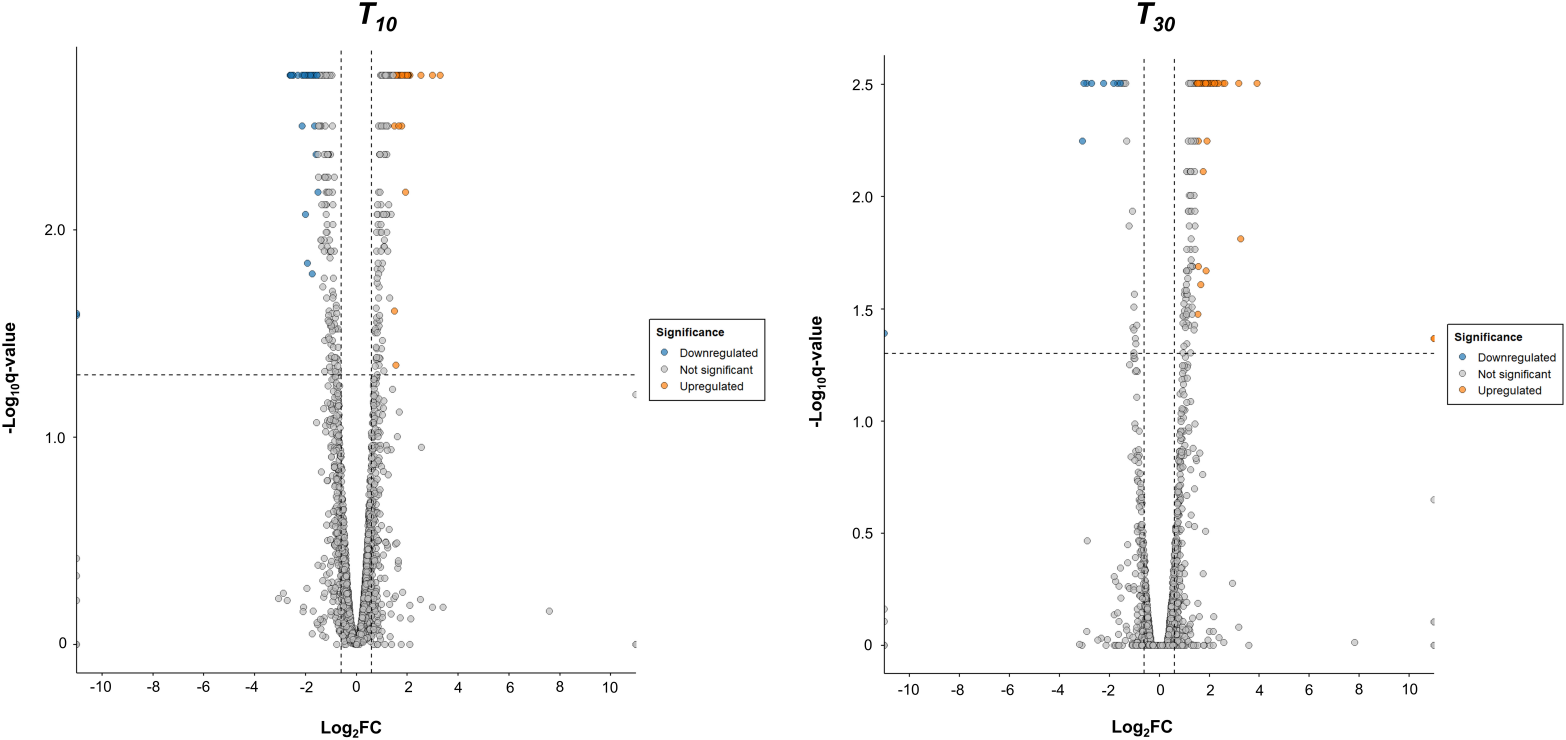
Volcano plot for differentially expressed genes (DEGs) in *Pseudomonas syringae* pv. tomato strain DAPP-PG 215 according to fold change (|Log_2_-Fold Change| > 1.5) and significance (*q*-value < 0.05) after 10 min (*T*_10_, left) and after 30 min (*T*_30_, right) of Argirium-SUNcs exposure. Each dot represents an individual gene: Turquoise dots represent the downregulated DEGs, pink dots represent the upregulated DEGs, and gray dots represent not differentially expressed genes.

Following RNA sequencing, quality metrics, including the total number of reads mapped and the sequencing depth, were assessed for each sample (Supplementary Table 1). As a next step, principal component analysis (PCA) of the data revealed that the first two principal components (PC1 and PC2) accounted for 52% of the total variance (31 and 21%, respectively; Supplementary Figure 1). Control replicates sampled after 10 min (C10) and 30 min (C30) from Argirium-SUNCs treatment formed tight clusters, indicating consistent expression profiles and low within-group variability. In contrast, the replicates of the treated groups sampled at 10 min (T10) and 30 min (T30) exhibited greater dispersion, with the samples showing a wider spread. This increased variability suggests either heterogeneous transcriptional responses to the treatment or greater biological variability, that may also be associated with the reduced sequencing depth observed in these samples (Supplementary Table 1). Despite the increased spread within the treated groups, clear separation was observed between control and treated samples, as well as between the two time points, further supporting a distinct treatment effect on gene expression profiles. Differential gene expression analysis using Cuffdiff revealed a total of 282 genes for *T*_10_ and 145 for *T*_30_ (*p* < 0.05), respectively, that were differentially expressed. Further filtering for absolute Log_2_-Fold Change > 1.5 revealed 78 DEGs in Argirium-SUNCs-treated cells harvested after 10 min (Supplementary Table 3), of which 35 (45%) genes were higher and 43 (57%) lower expressed than in the controls. In cells harvested after 30 min, the expression profiles of 66 genes were marked as significant (Supplementary Table 4), of which 57 (87%) genes were more expressed and 9 (13%) less expressed (Figure 1). Comparing the two timepoints, 21 shared DEGs were obtained, of which 18 genes were higher and three lower expressed as in control cultures (Table 1).

### 3.2 Functional analysis of DEGs

For a better understanding of the role of the genes involved in the first response of *P. syringae* pv. tomato strain DAPP-PG 215 to Argirium-SUNCs, functional classes were determined for the differentially expressed genes using gene ontology (GO) analysis. After 10 min of nanoparticles exposure, 243 GO terms related to biological process (68 identifiers for 49 genes), cellular component (46 identifiers for 59 genes), and molecular function (116 identifiers for 57 genes) categories were assigned (Supplementary Table 3). In biological process, transmembrane transport [GO:0055085] (6 genes), proteolysis [GO:0006508] (4 genes) and protein transport [GO:0015031] (3 genes) were the most represented subcategories. Plasma membrane [GO:0005886] (16 genes), cytosol [GO:0005829] (7 genes) and membrane [GO:0016020] (6 genes) were the three main sites in cellular component. Metal ion binding [GO:0046872] (10 genes), DNA-binding transcription factor activity [GO:0003700] (5 genes) and zinc ion binding [GO:0008270], ATP binding [GO:0005524] and ATP hydrolysis activity [GO:0016887] (4 genes each) were, on the other hand, the most frequently found terms in the molecular function category.

After 30 min, a total of 124 GO terms were assigned to the three main categories (21 in biological process, 30 in cellular component, and 36 in molecular function). No recurring identifiers were observed in biological process terms (Supplementary Table 4). In the cellular component category, however, plasma membrane [GO:0005886] (13 genes), cytoplasm [GO:0005737] (4 genes), membrane [GO:0016020], and cell outer membrane [GO:0009279] (3 genes each) were the most frequently encountered labels. Finally, metal ion binding (4 genes), ATP binding [GO:0005524], ATP hydrolysis activity [GO:0016887], and zinc ion binding [GO:0008270] (3 genes each) were found to be the most determined molecular functions.

### 3.3 Gene ontology and local network cluster enrichment

**FIGURE 2:**
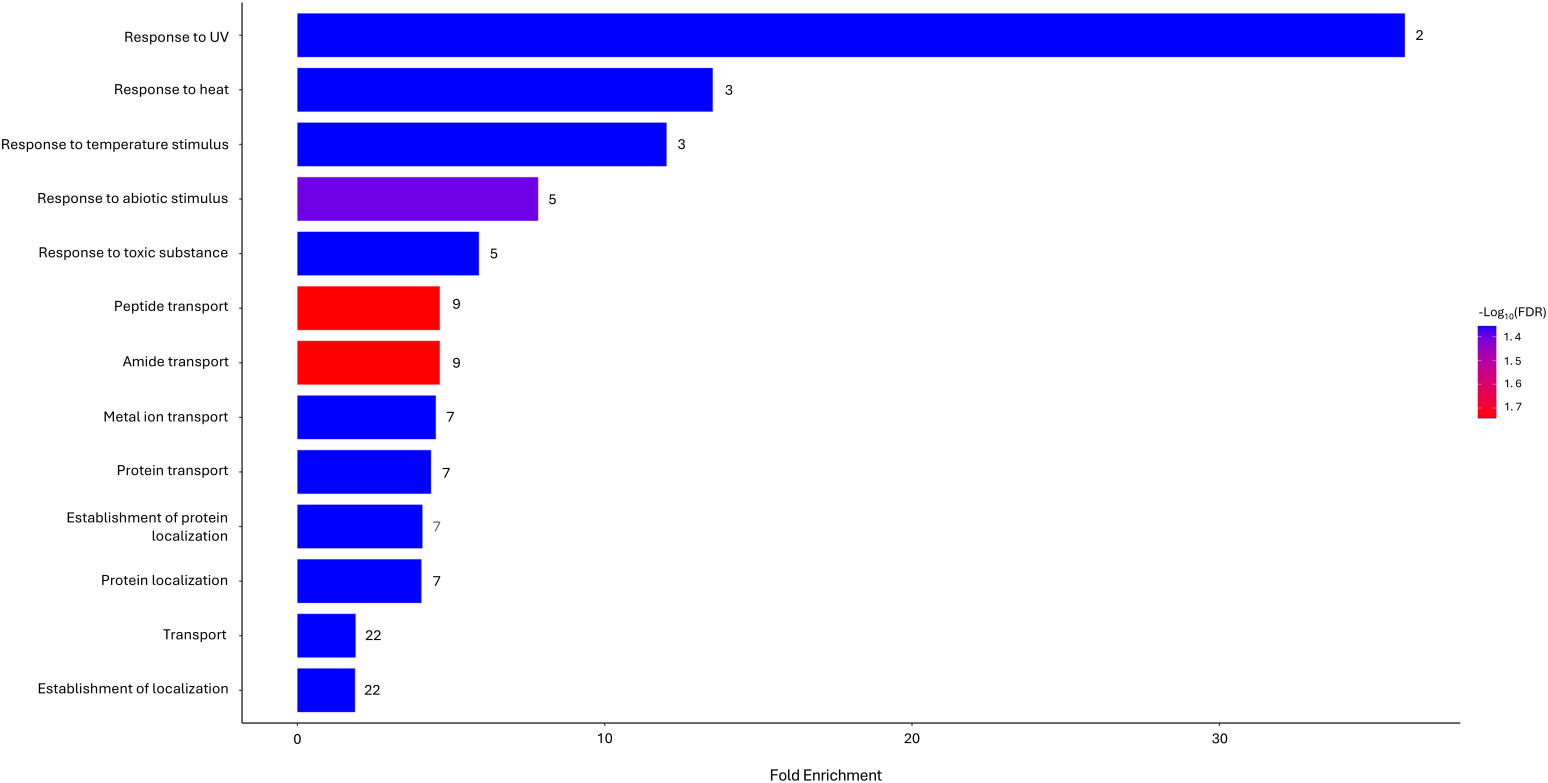
Biological processes enriched in Gene Ontology (GO) functional categories with *p*-value (FDR) < 0.05 in Argirium-SUNCs-treated group after 10-min exposure.

The functional enrichment analysis of differentially expressed gene sets revealed which responses, areas, and structures were significantly affected following exposure to silver nanoparticles. At time point *T*_10_, 13 different pathways associated with the GO biological process database were estimated to be among the most influenced by the action of Argirium-SUNCs. Of these, “Response to UV,” “Response to heat,” “Response to temperature stimulus,” “Response to abiotic stimulus,” and “Response to toxic substance” showed the highest fold enrichment (Figure 2). In the remaining significant entries, there was a notable presence of pathways related almost exclusively to transporters, specifically those involved in the cell migration of peptides, amides, metal ions, and proteins. Instead, after 30 min, only a significant involvement of the “Integral component of membrane” and “Intrinsic component of membrane” categories (*n* = 23, FDR = 4.40^–2^ for both assignments) in cellular component was reported.

**FIGURE 3:**
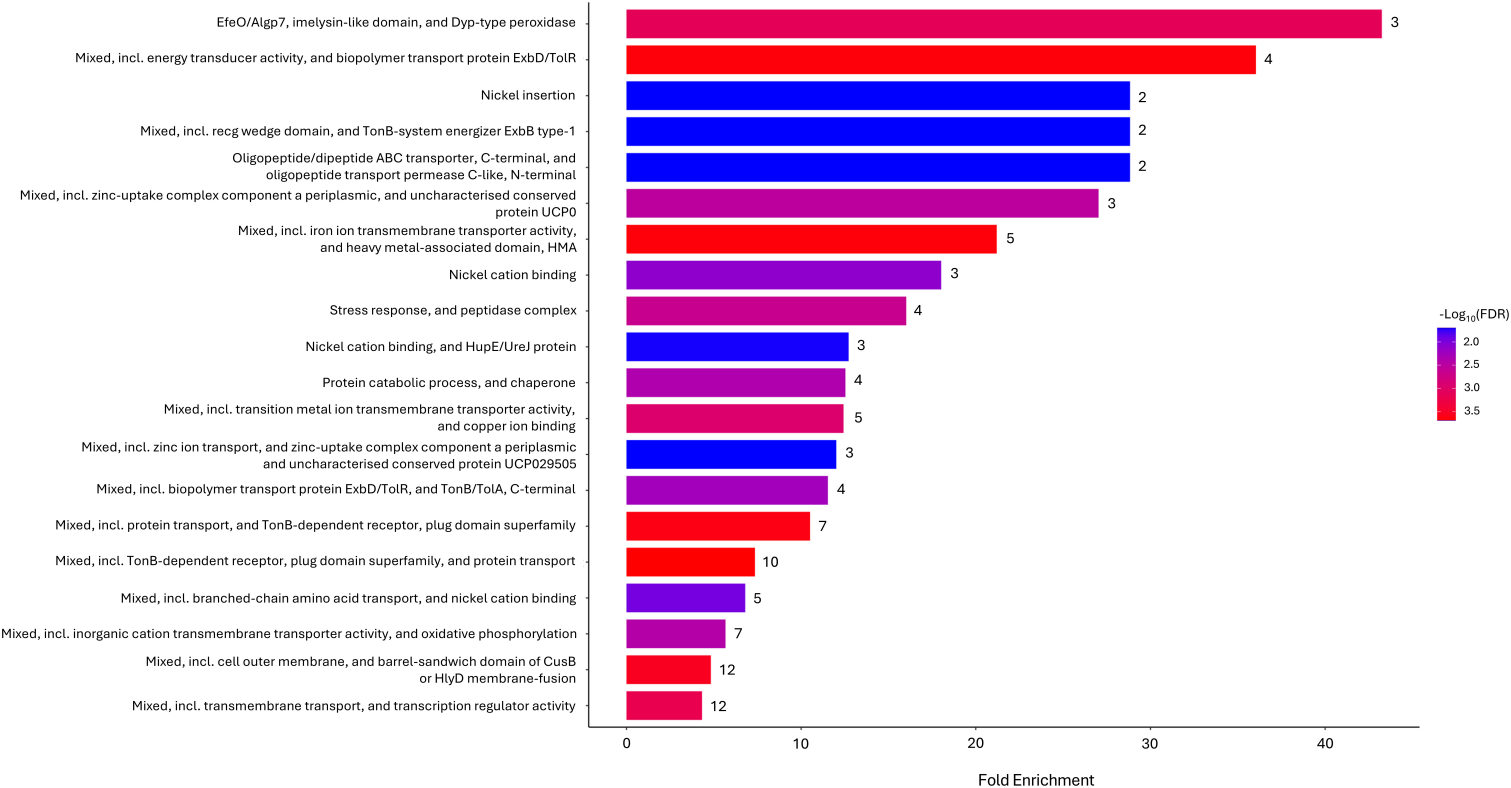
STRING local network cluster enriched in Gene Ontology (GO) functional categories with *p*-value (FDR) < 0.05 in Argirium-SUNCs-treated group after 10-min exposure.

**FIGURE 4:**
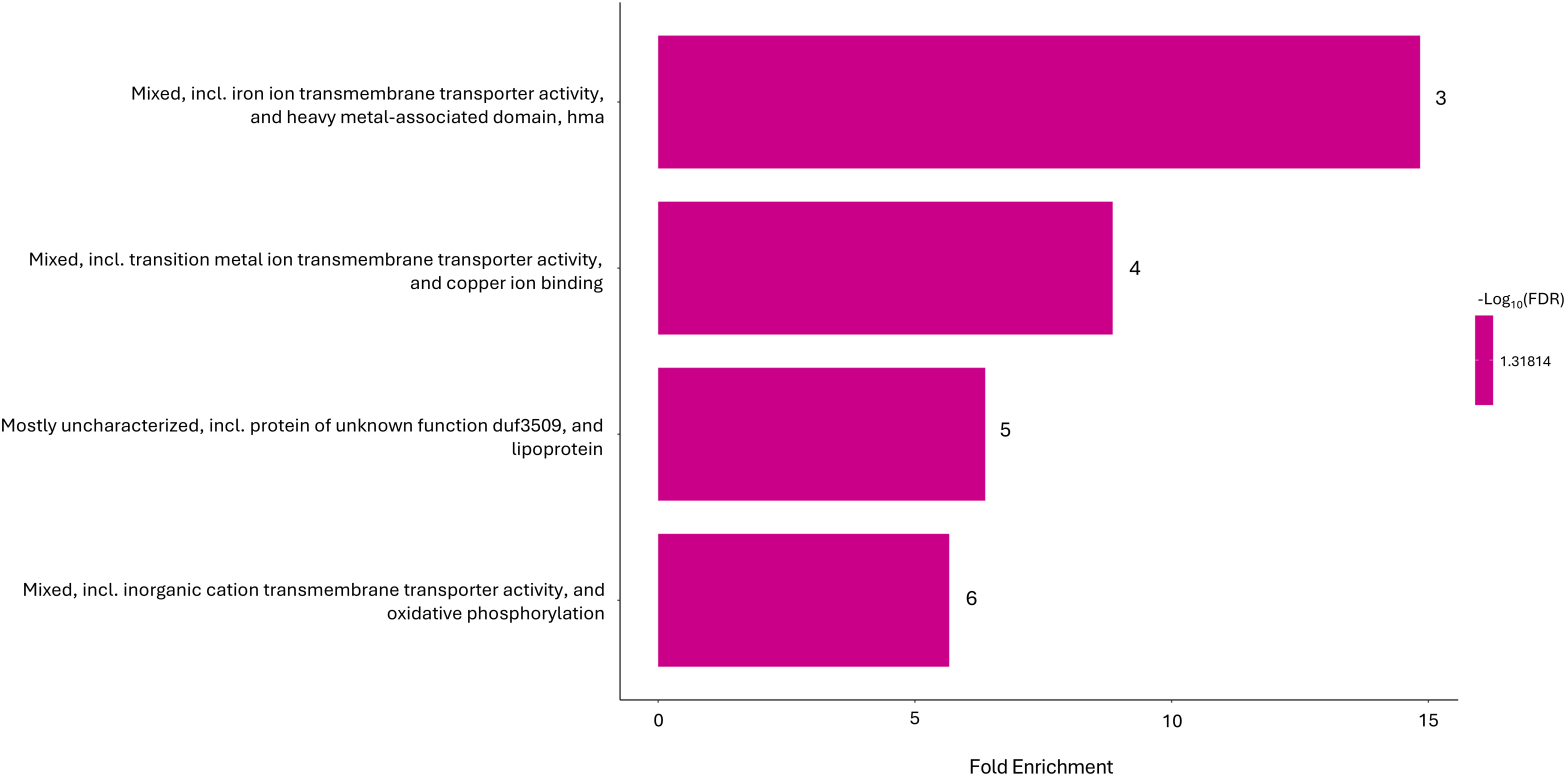
STRING local network cluster enriched in Gene Ontology (GO) functional categories with *p*-value (FDR) < 0.05 in Argirium-SUNCs-treated group after 30-min exposure.

Regarding the functional enrichment of the local network clusters, after 10 min of exposure, 20 significantly expressed pathways were identified (Figure 3). In this case as well, a predominant distribution of categories related to transporters was observed (50%). Additionally, several entries were associated with the Tol-Pal and TonB-ExbB-ExbD systems (25%). Three of the pathways present at *T*_10_, namely “Mixed, including iron ion transmembrane transporter activity, and heavy metal-associated domain, hma,” “Mixed, including transition metal ion transmembrane transporter activity, and copper ion binding,” and “Mixed, including inorganic cation transmembrane transporter activity, and oxidative phosphorylation” were also estimated to be significant after 30 min of exposure, along with a fourth category: “Mostly uncharacterized, including protein of unknown function DUF3509, and lipoprotein” (Figure 4).

### 3.4 KEGG pathway enrichment

The KOBAS-i pathway enrichment analysis mapped differentially expressed genes at *T*_10_ onto 31 KEGG pathways (Supplementary Table 4). Although raw *p*-values suggested potential enrichment for some pathways such as “Quorum sensing” give genes out of 95; raw *p* = 0.0046), “2-Oxocarboxylic acid metabolism” (2/25; raw *p* = 0.0356), and “Arginine biosynthesis” (two genes out of 25; raw *p* = 0.0356), none of these remained statistically significant after multiple testing correction (FDR), with adjusted *p* ≥ 0.14.

At the 30-min time point, 11 KEGG pathways were detected (Supplementary Table 6). However, even in this case no pathway resulted in statistical significance.

### 3.5 Quantitative RT-PCR validation

**FIGURE 5:**
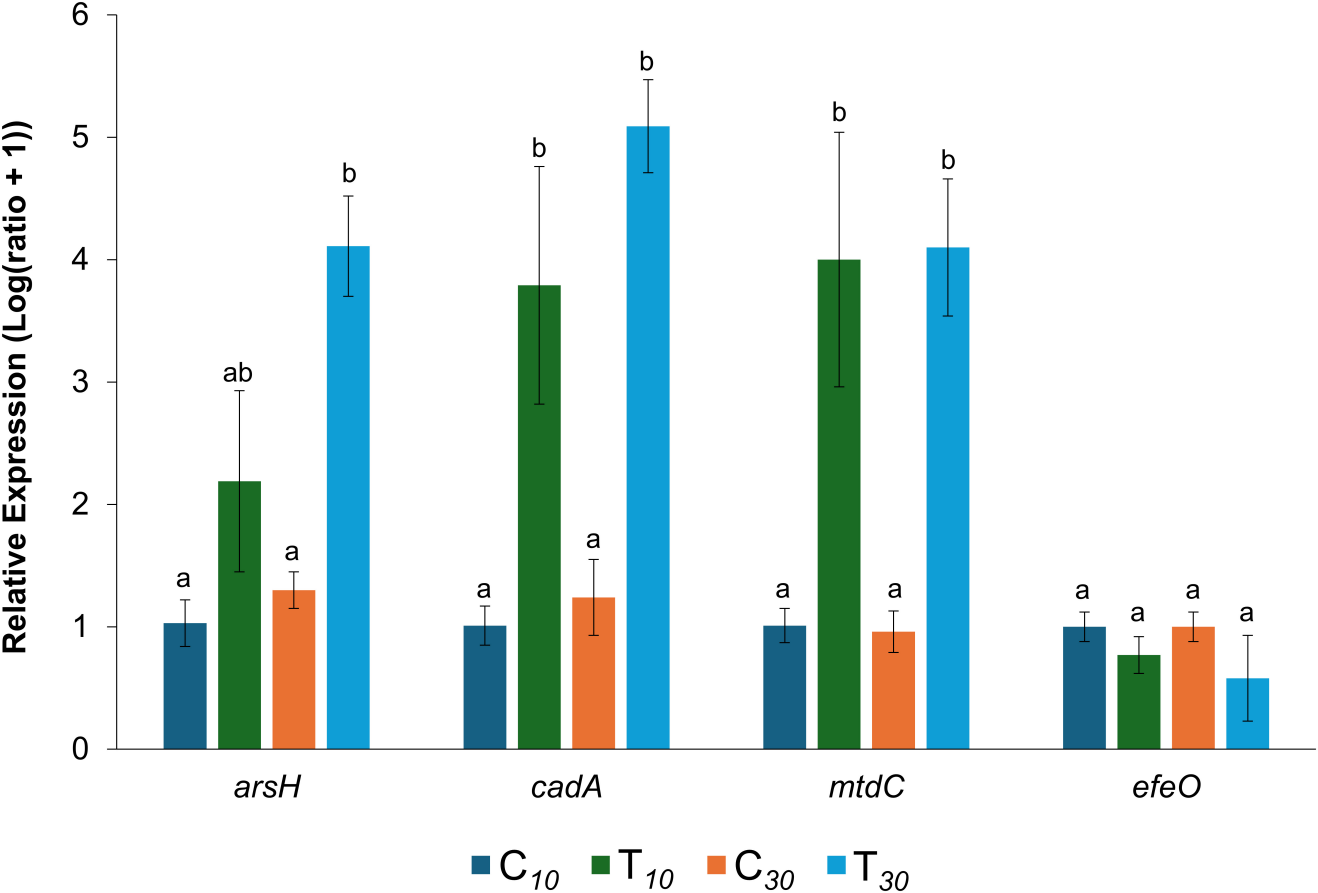
Relative expression of four genes, three upregulated (*arsH*, *cadA* and *mtdC*) and one downregulated (*efeO*), selected for qPCR validation on RNA-Seq results, compared to the control. C10 = control, untreated, sampled after 10 min; T10 = control, treated with Argirium-SUNcs, sampled after 10 min; C30 = ; control, untreated, sampled after 30 min T30 = control, treated with Argirium-SUNcs, sampled after 10 min. Data are means of nine replicates (three biological, three technical) ± SE. Columns with different letters are significantly different (*p* ≤ 0.05; Tukey’s multiple comparison test).

**FIGURE 6:**
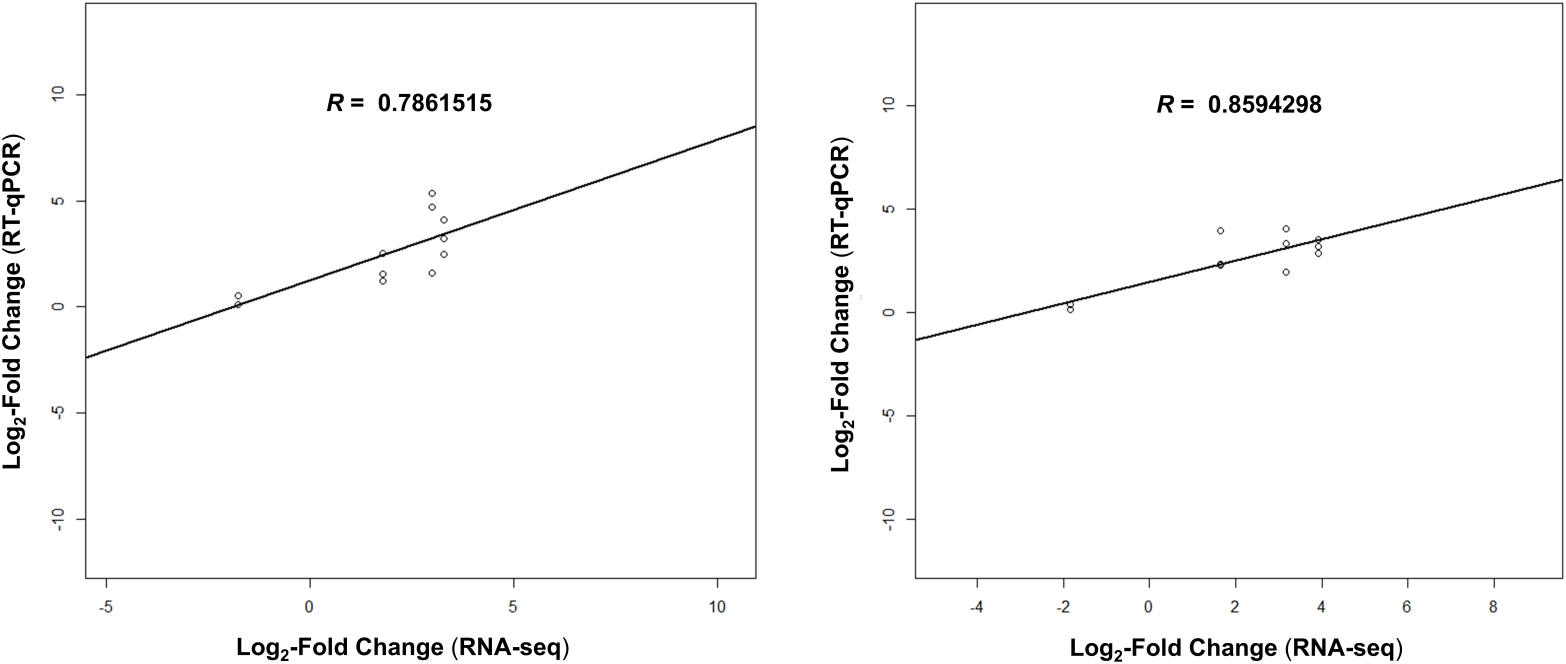
Correlations between Log_2_-Fold Change obtained by RNA-Seq and qPCR validation for samples collected after 10 min (*T*_10_, left) and 30 min (*T*_30_, right) for each biological replicate. Pearson’s *R* values are indicated in the figure. *P*-values: 0.0006 (*T*_10_) and 0.0003 (*T*_30_).

Differentially expressed gene results from RNA-Seq experiment were validated by a RT-qPCR test. Three higher expressed genes (*arsH*, *cadA* and DAPPPG215_01280) and one lower expressed gene (*efeO*) were chosen for the comparison with two reference housekeeping genes (*recA* and *rpoD*). The relative expression of the tested genes in both the groups (control and Argirium-SUNCs), at the different time-points (10 and 30 min of exposure to water/Argirium-SUNCs) was submitted to ANOVA and Tukey’s multiple comparison tests (*p* = 0.05) (Figure 5). Similar patterns of DEGs were observed in RT-qPCR and RNA-Seq analyses in terms of expression, although differences between control and Argirium-SUNCs-treated samples were not statistically significant for the less expressed gene *efeO*. However, when relative expression deduced with RT-qPCR, transformed in LFC, was compared with the results of the RNA-Seq expression analysis, a significant positive correlation was observed (correlation coefficients for 10-min exposure and 30-min exposure = 0.79–0.86, *p* = 0.002–0.001, respectively), suggesting the reliability and quality of the RNA-Seq analysis (Figure 6).

## 4 Discussion

The aim of this work was to study the transcriptome of *P. syringae* pv. tomato strain DAPP-PG 215 after a 10 to 30-min exposure to a subinhibitory concentration of Argirium-SUNCs in order to better understand the first response of the pathogen to silver nanoparticles (AgNPs).

Principal component analysis (PCA) and sequencing quality controls revealed increased variability exclusively in Argirium-SUNC–treated samples, whereas untreated controls clustered closer and a clear separation between the two time points can be observed for both conditions. This indicates that the treatment, rather than technical factors, introduced biological heterogeneity into the transcriptomic response. A plausible hypothesis is that Argirium-SUNCs, which contain atypical silver oxidation states (Ag^2+^/Ag^3+^), may generate non-uniform levels of oxidative and membrane stress across the bacterial population, causing variable degrees of RNA damage, membrane perturbation, and activation of stress pathways.

Despite this variability, functional analyses highlighted coherent and significant differential expression of genes localized to the plasma membrane at both time points. Regarding pathways, although the KEGG pathway analysis following Argirium-SUNCs exposure did not resulted in any significant enrichment, likely due to the limited number of differentially expressed genes detected, functional enrichment of GO terms and analysis of STRING local network clusters revealed a significant involvement of abiotic stress response and transporter proteins related to energy metabolism and metal ion homeostasis. These observations are consistent with previous studies: AgNPs would likely accumulate on the plasma membrane, not only causing a degeneration of the proton motive force but also forming pores on the cell surface (Singh et al., 2016; Molina-Hernandez et al., 2021). Then, once the membrane is damaged, AgNPs would penetrate into the cytoplasm, binding to enzymes with a thiol (sulfhydryl) groups, inactivating them and leading to hydroxyl radical generation due to iron leakage (Gordon et al., 2010; Khalandi et al., 2017). Importantly, silver can compete with other metals for binding sites in proteins, like copper (Eckhardt et al., 2013; Kozlowski et al., 2013). In this study, Argirium-SUNCs seem to interact with various metal-binding proteins, including those associated with cadmium, iron, nickel, copper, and zinc, as well as metalloids such as arsenic. This characteristic may be due to the fact that Argirium-SUNCs contain silver ions in oxidation states Ag^2+^/Ag^3+^, which could confer a variable and distinct transcriptional response compared with other silver nanoparticles and silver ions in general.

At 10-min timepoint, the most significantly enriched functional categories, based on fold change, were those related to biological processes involved in the response to various abiotic stimuli. Genes involved were DAPPPG215_03880 (*clpB*; LFC = 1.51), DAPPPG215_10650 (*efeO*; LFC = −1.58), DAPPPG215_10660 (LFC = −1.82), DAPPPG215_16600 (*hopI1*; LFC = −1.91) and DAPPPG215_21795 (*lon−2*; LFC = 1.62). Proteins ClpB and Lon are members of the AAA + protein family (ATPases associated with diverse cellular activities). When expressed at higher levels, these proteins have been associated with bacterial resistance to osmotic stress due to their critical roles in protein folding, repair, and degradation (Xue et al., 2019; Mason et al., 2020; Alam et al., 2021). HopI1, on the other hand, is an essential virulence factor of *P. syringae* that was correlated with the ATP-mediated hydrolysis of Hsp70, the main heat shock protein (Jelenska et al., 2010; Ghazaei, 2017). Therefore, it is plausible that the increased expression of the *clpB* and *lon-2* genes and the lower expression of *hopI1* shortly after the exposure to AgNPs is due to an attempt by *P. syringae* pv. tomato to resist and reduce intracellular osmotic stress. In addition, DAPPPG215_10650 and DAPPPG215_10660 are genes involved in iron uptake DAPPPG215_10660 is annotated as *efeO*, a periplasmic iron-binding protein belonging to the EfeUOB iron uptake system, which is crucially linked to oxidative stress responses (Cornelis et al., 2011). Similarly, DAPPG215_10650 is a metalloprotein located downstream of the *efeUOB* operon provisionally annotated as an inactive ferrous ion transporter periplasmic EfeO. It could potentially be analogous to the additional EfeO-like protein Algp7 as described by Temtrirath et al. (2017).

In the functional category “response to toxic substance,” along with three genes associated with EfeO (DAPPPG215_10660, DAPPPG215_10655, DAPPPG215_10650), there are two efflux pumps involved in multidrug resistance, DAPPPG215_03455 and DAPPPG215_17665. DAPPPG215_03455, annotated as a Bcr/CflA family efflux transporter, is upregulated (LFC = 2.23). In contrast, DAPPPG215_17665, annotated as AcrA, is downregulated (LFC = −2.07). This differential expression suggests that the bacterium may rely on different efflux systems depending on the specific nature of the stressor, and that the regulatory patterns of these efflux pumps may vary in response to the specific stress condition.

The STRING local network cluster-based enrichment highlights that transport and metal homeostasis genes are central to the bacterial response. Multiple hits addressed a strong enrichment of TonB/ExbB/TBDR genes, revealing a strong conditioning of the TonB system following exposure to Argirium-SUNCs. Seven genes (DAPPPG215_15900, DAPPPG215_11940, DAPPPG215_15895, DAPPPG215_00250, DAPPPG215_00635, DAPPPG215_00240, DAPPPG215_03035), all less expressed, are linked to the activity of the TonB system, a complex comprising the cell membrane proteins TonB, ExbB, and ExbD and responsible for the uptake of iron and other substances from the outer membrane, utilizing energy derived from the proton motive force (Higgs et al., 2002; Celia et al., 2016; Fujita et al., 2019). While Slavin et al. (2021) reported upregulation of TonB in *Pseudomonas aeruginosa* exposed to sublethal concentrations of lignin-capped AgNPs, the reduced expression observed here may result from stress associated with membrane depolarization (Molina-Hernandez et al., 2021), a mechanism already recognized as the basis for the antimicrobial action of AgNPs. Recent investigations have also shown that silver and zinc oxide nanoparticles (ZnONPs) can directly bind to TonB-dependent transporters (Binert-Kusztal et al., 2025; Shakerdarabad et al., 2024), confirming the centrality of the TonB system in understanding the mechanism of action of metal nanoparticles. However, further research is needed to elucidate whether the observed regulatory changes and the structural interactions between nanoparticles and TonB transporters are mechanistically linked. Other aspects to clarify the role of TonB concern the presence, among the less expressed genes in our study, of the gene coding for ZnuA (DAPPPG215_17775), a periplasmic Zn^2+^-binding protein that has been associated with TonB (Mazzon et al., 2014; Pederick et al., 2015), and the ability of this system to directly affect the production of flagellin (Dong et al., 2023), which Panáček et al. (2018) identified as a mechanism of bacterial resistance to AgNPs.

At 30-min exposure, two main groups of genes were observed. The first group consisted of mostly uncharacterized proteins, including lipoproteins and DUF3509-containing proteins, likely associated with the plasma membrane. The second group, corresponding to the first three categories, confirmed the strong representation of genes involved in metal transport and associated processes. In particular, genes such as DAPPPG215_01375, DAPPPG215_03455, DAPPPG215_10650 and DAPPPG215_26360 were overrepresented. DAPPPG215_01375, DAPPPG215_03455, and DAPPPG215_26360 are three efflux pumps that were all upregulated both at 10- and 30-min exposure. The Bcr/CflA family efflux transporter DAPPPG215_03455, the cadmium-translocating P-type ATPase encoding gene *cadA* (DAPPPG215_26360), and the arsenical efflux pump membrane protein ArsB (DAPPPG215_01375) can be associated to detoxification mechanisms against heavy metals and xenobiotics. Multidrug resistance (MDR) proteins like DAPPPG215_03455 are translocators deputed to extrude xenobiotics and drugs. They are known to have a broad spectrum of efficacy, and their activity has also been associated with pH stress (Edgar and Bibi, 1997; Hayes et al., 2006; Radi et al., 2022). Furthermore, the role mediated by efflux pumps eliminating Ag ions as demonstrated by Li et al. (1997) was already observed in silver-resistant mutants of *E. coli* (Stabryla et al., 2021).

CadA instead was previously shown to confer resistance to cadmium, zinc and lead (Schwager et al., 2012). However, although the majority of P-type pumps show distinct substrate specificities and can be classified as efflux pump involved in detoxification of monovalent metals (such as Cu^+^ and Ag^+^) or efflux pumps involved in detoxification of the divalent metals (such as Cd^2+^, Zn^2+^, Pb^2+;^ (Rensing et al., 2000; Sharma et al., 2000; Argüello et al., 2007; Lewinson et al., 2009), Gualdi et al. (2022) demonstrated that CadA of *Burkholderia cenocepacia* is a P-type pump involved in the detoxification of Ag^+^.

**FIGURE 7:**
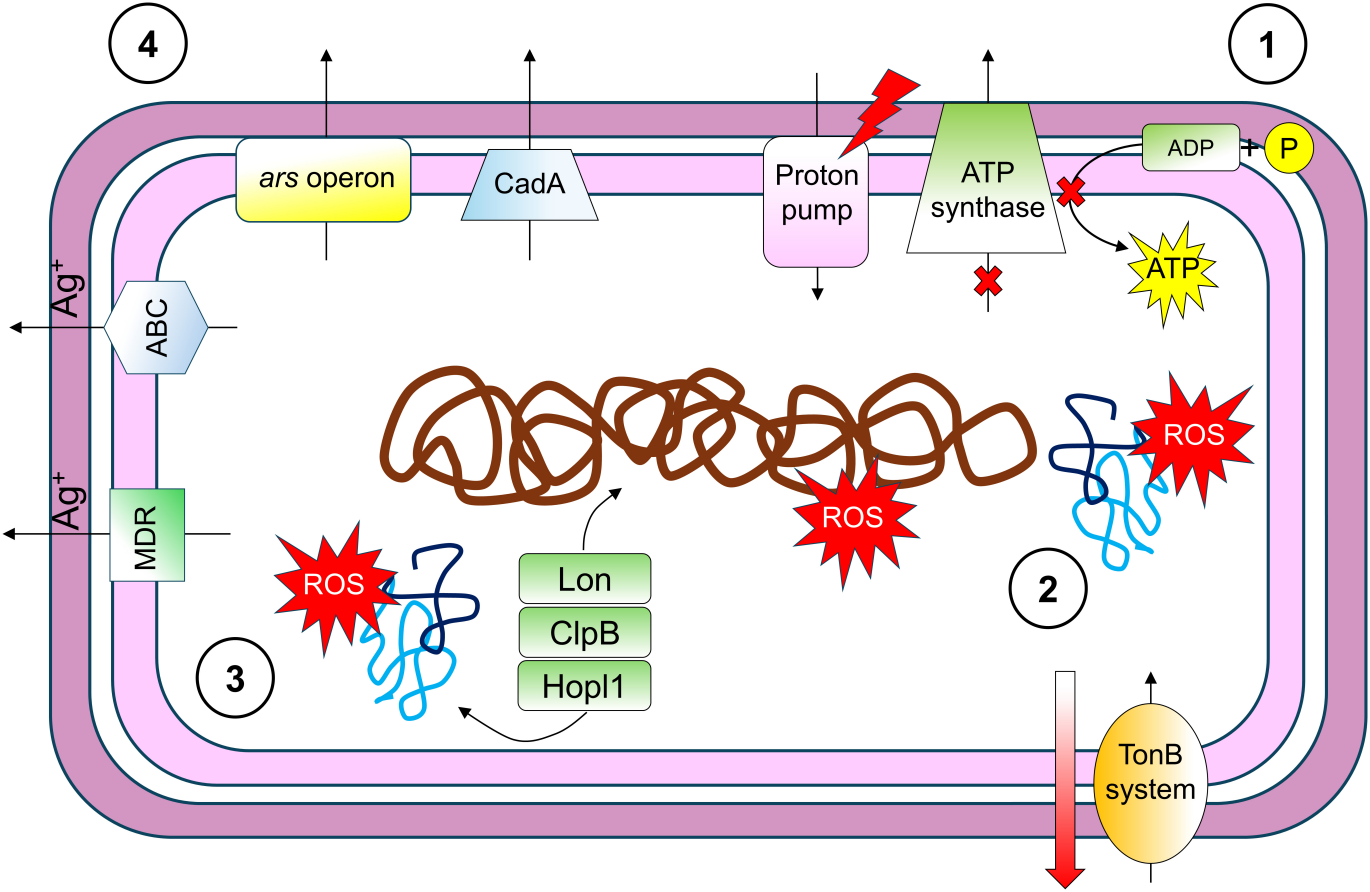
Organization of the arsenate-resistance operon (*ars*) in *Pseudomonas syringae* pv. tomato DAPP-PG 215. *arsR1*, arsenic resistance transcriptional regulator; *arsB*, arsenical pump membrane protein; *arsC*, arsenate reductase; *arsH*, arsenical resistance protein; *arsO*, ArsO family NAD(P)H-dependent flavin-containing monooxygenase; *MFS transporter*, Major Facilitator Superfamily transporter. Values reported under the genes indicate the Log_2_-Fold Change expression compared to the untreated control after 10 and 30 min after the treatment with Argirium-SUNCs.

Moreover, Argirium-SUNCs are AgNPs characterized by rare oxidative states (Ag^2+/3+^, Orfei et al., 2024) and therefore it is possible that a unique feature of the Argirium-SUNCs is involved in provoking the specific response. But the reduction of ionic Ag to the less toxic Ag^0^, mediated by an arsenate reductase could be another defense mechanism (Stabryla et al., 2021). We revealed that the main genes of the arsenate (*ars*) operon are marked expressed in Argirium-SUNCs treated *P. syringae* pv. tomato cells (Figure 7). As the only metal present in Argirium-SUNCs is silver, this metal should be used as a substrate of the proteins encoded by the *ars* operon. Similar to the mechanism described by Kumari and Jagadevan (2016), we can argue that ionic Ag is reduced to Ag^0^ by the arsenate reductase (*arsC*), a process in which glutathione serves as the electron donor. According to this scenario, ArsB, an arsenical pump membrane protein, should possibly facilitate the efflux of Ag^0^.

## 5 Conclusion

**FIGURE 8:**
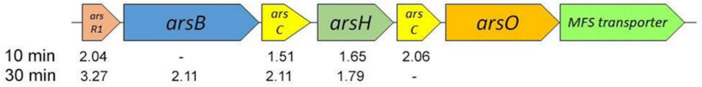
Graphic representation of *Pseudomonas syringae* pv. tomato DAPP-PG 215 response to silver nanoparticles (AgNPs) exposure. AgNPs penetrate through the outer membrane and accumulate in the inner membrane, affecting both membrane potential and proton motive force, causing membrane disruption, cytoplasmic leakage, and a decrease in ATP level (1). As AgNPs progressively accumulate in the cytoplasm, they impair the active transport of iron compounds across the outer membrane via the TonB system and enhance reactive oxygen species (ROS) generation, which leads to DNA damage and protein denaturation (2). Genes involved in osmotic stress regulation are upregulated as part of a detoxification response (3), in which numerous transporters responsible for the export of xenobiotics and heavy metals (e.g., ArsC, CadA) also play a key role (4).

Silver nanoparticles (AgNPs) are notably a potent antimicrobial agent effective against a wide range of pathogenic microorganisms. As far as we know, this work represents the first report on the transcriptomic analysis of a plant pathogenic bacterium, *P. syringae* pv. tomato strain DAPP-PG 215, challenged with AgNPs (Argirium-SUNCs). The results presented in this study and summarized in Figure 8 revealed the crucial role of enzymes and transporters involved in oxidative stress, detoxification of heavy metals and xenobiotics in the first response to Argirium-SUNCs exposure, suggesting an attempt of defense in *P. syringae* pv. tomato to escape cell lysis due to osmotic stress and mechanical damage following the entrance of AgNPs.

Our data indicate that the earliest transcriptional response is dominated by two main processes: the downregulation of metal-binding and TonB-dependent uptake systems, and the upregulation of detoxification pathways involved in heavy metal extrusion and redox balance. The strong and consistent repression of TonB/ExbB/ExbD components suggests that AgNPs profoundly affect metal homeostasis and energy-dependent uptake of essential metals, likely reflecting membrane depolarization and possibly as a consequence of competition between silver ions and natural metal-binding sites. Nevertheless, given the contrasting results regarding this downregulation, TonB remains a central element to investigate further in elucidating the mechanism of action of silver nanoparticles.

In parallel, the marked induction of *cadA*, the *ars* operon, and broad-spectrum efflux pumps indicates an immediate activation of mechanisms dedicated to extrusion or reduction of toxic metal species, including silver ions. This coordinated response supports a model in which *P. syringae* pv. tomato DAPP-PG 215 rapidly restricts metal entry while enhancing detoxification to counteract AgNP-induced stress. Nevertheless, further investigations will be required to determine how these responses integrate into the complete antimicrobial mechanism of silver nanoparticles, including the potential contribution of their distinct oxidation states, and whether they differ from responses triggered by ionic silver or other heavy metals.

## Data Availability

Raw reads for all samples of this study are available at NCBI Gene Expression Omnibus (GEO) database (accession number: GSE308472).

## Footnotes

1
http://www.ncbi.nlm.nih.gov/
