# Correction: Transcriptome analysis of *Pseudomonas syringae* pv. tomato DAPP-PG 215 in response to silver nanoparticles exposure

**DOI:** 10.3389/fmicb.2026.1809727

**Published:** 2026-03-03

**Authors:** Benedetta Orfei, Joël F. Pothier, Luca Scotti, Antonio Aceto, Chiaraluce Moretti, Roberto Buonaurio, Theo H. M. Smits

**Affiliations:** 1 Department of Agricultural, Food and Environmental Sciences, University of Perugia, Perugia, Italy; 2 Environmental Genomics and Systems Biology Research Group, Institute of Natural Resource Sciences (IUNR), Zurich University of Applied Sciences (ZHAW), Wädenswil, Switzerland; 3 IIS “Alessandrini-Marino”, Teramo, Italy; 4 Retired, Teramo, Italy

**Keywords:** heavy metal resistance, RNA-seq, nanoparticle, antibacterial mechanism, biocontrol

An incorrect Funding statement was provided.

The correct Funding statement is below:

“The author(s) declared that financial support was received for this work and/or its publication. JP and TS were supported by the Department of Life Sciences and Facility Management of the Zurich University of Applied Sciences (ZHAW) inWädenswil. The EDGAR platform was funded by the BMBF grant FKZ031A533 within the de.NBI network. CM and RB were supported by the project “Innovative farm strategies that integrate sustainable N fertilization, water management and pest control to reduce water and soil pollution and salinization in the Mediterranean (Safe-H2O-Farm).” The Safe-H_2_O-Farm project is part of the PRIMA Programme, supported by the European Union, with co-funding from the Italian Ministry of University and Research (MUR).”

The original version of this article has been updated.

