# Supplementary material for: Transcriptome analysis of Pseudomonas syringae pv. tomato DAPP-PG 215 in response to silver nanoparticles exposure
Source: Front Microbiol. 2026 Jan 6;16:1714857. doi: 10.3389/fmicb.2025.1714857 (PMC12816370; doi:10.3389/fmicb.2025.1714857)
Supplement: Supplementary file 1 [file Data_Sheet_1.docx]

# Supplementary Figures


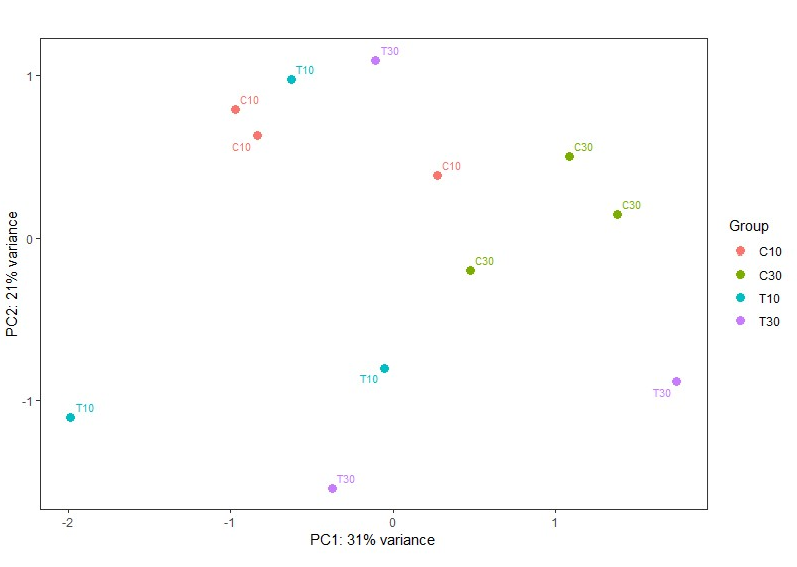


**Supplementary Figure 1.** Principal component analysis (PCA) of samples based on rlog-transformed raw counts. The first two principal components (PC1: 31%, PC2: 21%) together explain 52% of the total variance. Samples are colored according to experimental groups: C10, C30, T10, and T30.

# Supplementary Tables

**Supplementary Table S1.** Summary of RNA-Seq quality metrics. Total reads (raw reads generated), mapped reads (reads successfully aligned to the reference), mapping percentage (% of total reads that were mapped), and average depth (average number of reads per transcript) are provided for each biological replicate.

| **Sample** | **Total Reads** | **Mapped Reads** | **Mapping Percent (%)** | **Average Sequencing Depth** |
| --- | --- | --- | --- | --- |
| C10_1 | 21.592.288 | 18.974.130 | 87.9 | 5.4 |
| C10_2 | 20.828.140 | 18.566.072 | 89.1 | 5.3 |
| C10_3 | 15.623.078 | 13.924.918 | 89.1 | 3.9 |
| C30_1 | 19.738.740 | 17.553.247 | 88.9 | 5.0 |
| C30_2 | 15.378.752 | 13.738.582 | 89.3 | 3.9 |
| C30_3 | 18.635.740 | 16.588.178 | 89.0 | 4.7 |
| T10_1 | 18.357.244 | 16.368.777 | 89.2 | 4.6 |
| T10_2 | 15.279.464 | 13.479.690 | 88.2 | 3.9 |
| T10_3 | 16.624.484 | 14.813.767 | 89.1 | 4.2 |
| T30_1 | 17.198.458 | 15.380.618 | 89.4 | 4.3 |
| T30_2 | 14.959.550 | 13.427.770 | 89.8 | 3.8 |
| T30_3 | 14.934.892 | 13.255.402 | 88.8 | 3.8 |

**Supplementary Table S2.** List of the primers used for RNA-seq data qPCR validation. Reference housekeeping genes are marked with an asterisk (*).

| **Gene target** | **Locus tag** | **Primer name** | **Primers (5’-3’)** | **PCR *T_m_* (°C)** |  |
| --- | --- | --- | --- | --- | --- |
|  |  |  |  |  |  |
| arsenical resistance protein *arsH* | DAPPPG215_01365 | arsH For arsH Rev | CGAACGTACACACCGACCT  CGATGGCACTCAGCTTGTCG | 63 |  |
|  |  |  |  |  |  |
| cadmium-translocating P-type ATPase *cadA* | DAPPPG215_26360 | cadA For  cadA Rev | CGTGAAGGAAACCAGCCGTG  CGATGGCACTCAGCTTGTCG | 63 |  |
| efflux RND transporter permease subunit | DAPPPG215_01280 | mtdC For  mtdC Rev | GACCGCACAAGACGCCAAAA  CTGACCCCGACCATCTGCAT | 61 |  |
| iron uptake system component *efeO* | DAPPPG215_10650 | efeo For  efeO Rev | GCAGGCGGGCTGTTCTACTA  GGAGCGGTTGACGATACGGA | 61 |  |
| recombinase *recA* * | DAPPPG215_08130 | recA For  recA Rev | TAGAACTTCAGCGCGTTACC  GCCAACTGCCTGGTTATCT | 61 |  |
| RNA polymerase sigma factor *rpoD* * | DAPPPG215_02320 | rpoD For  rpoD Rev | GAAGTTGACGAAAGCTGGACCG  CGACGGTTGATGTCCTTGATCTC | 61 |  |
|  |  |  |  |  |  |

**Table S3.** List of differentially expressed genes (DEGs) in *Pseudomonas syringae* pv. tomato strain DAPP-PG 215 exposed to Argirium-SUNCs for 10 minutes (*T_10_*) with their UniProt ID, BLAST annotation and log_2_-Fold Change

| **Locus tag** | **Annotation** | **log_2_-Fold Change** | **Gene Ontology**  **(biological process)** | **Gene Ontology**  **(cellular component)** | **Gene Ontology**  **(molecular function)** |
| --- | --- | --- | --- | --- | --- |
| DAPPPG215_26360 | Cadmium-translocating P-type ATPase | 3.29433 |  | cytosol [GO:0005829] | DNA-binding transcription factor activity [GO:0003700]; double-stranded DNA binding [GO:0003690] |
| DAPPPG215_01280 | Efflux RND transporter permease subunit | 3.00279 |  | plasma membrane [GO:0005886] | ATP binding [GO:0005524]; ATP hydrolysis activity [GO:0016887]; ATPase-coupled monoatomic cation transmembrane transporter activity [GO:0019829]; cadmium ion transmembrane transporter activity [GO:0015086]; metal ion binding [GO:0046872]; P-type ion transporter activity [GO:0015662] |
| DAPPPG215_19955 | Protease HtpX (EC 3.4.24.-) (Heat shock protein HtpX) | 2.54452 |  | plasma membrane [GO:0005886] | monoatomic cation transmembrane transporter activity [GO:0008324]; xenobiotic transmembrane transporter activity [GO:0042910] |
| DAPPPG215_03455 | Bcr/CflA family efflux transporter | 2.10137 | proteolysis [GO:0006508] | plasma membrane [GO:0005886] | metalloendopeptidase activity [GO:0004222]; zinc ion binding [GO:0008270] |
| DAPPPG215_01360 | Arsenate reductase (EC 1.20.4.1) | 2.05579 | xenobiotic detoxification by transmembrane export across the plasma membrane [GO:1990961] | plasma membrane [GO:0005886] | sodium:proton antiporter activity [GO:0015385]; xenobiotic transmembrane transporter activity [GO:0042910] |
| DAPPPG215_26355 | Formimidoylglutamase, putative | 2.0408 |  |  | arsenate reductase (glutaredoxin) activity [GO:0008794] |
| DAPPPG215_01380 | Transcriptional regulator, ArsR family | 2.04047 |  |  |  |
| DAPPPG215_27315 | Phosphatidate cytidylyltransferase (EC 2.7.7.41) | 2.01534 |  |  | DNA-binding transcription factor activity [GO:0003700] |
| DAPPPG215_12780 | Alkyl hydroperoxide reductase subunit F | 2.00792 | CDP-diacylglycerol biosynthetic process [GO:0016024] | plasma membrane [GO:0005886] | phosphatidate cytidylyltransferase activity [GO:0004605] |
| DAPPPG215_25700 | ATP-dependent protease subunit HslV (EC 3.4.25.2) | 1.95709 | response to reactive oxygen species [GO:0000302] |  | flavin adenine dinucleotide binding [GO:0050660]; NAD binding [GO:0051287]; NADH-dependent peroxiredoxin activity [GO:0102039]; oxidoreductase activity, acting on a sulfur group of donors, NAD(P) as acceptor [GO:0016668] |
| DAPPPG215_07920 | Peptidase M10 metallopeptidase domain-containing protein | 1.95533 | proteolysis involved in protein catabolic process [GO:0051603] | HslUV protease complex [GO:0009376]; proteasome core complex [GO:0005839] | metal ion binding [GO:0046872]; threonine-type endopeptidase activity [GO:0004298] |
| DAPPPG215_07915 | Hydrolase, alpha/beta fold family | 1.94006 | proteolysis [GO:0006508] | extracellular matrix [GO:0031012] | metalloendopeptidase activity [GO:0004222]; zinc ion binding [GO:0008270] |
| DAPPPG215_13965 | UDP-glucose 6-dehydrogenase (EC 1.1.1.22) | 1.93389 | acylglycerol catabolic process [GO:0046464] | membrane [GO:0016020] | monoacylglycerol lipase activity [GO:0047372] |
| DAPPPG215_08170 | ferredoxin--NADP(+) reductase (EC 1.18.1.2) | 1.93235 | polysaccharide biosynthetic process [GO:0000271]; UDP-glucuronate biosynthetic process [GO:0006065] |  | NAD binding [GO:0051287]; UDP-glucose 6-dehydrogenase activity [GO:0003979] |
| DAPPPG215_06925 | Type 3 secretion system secretin (T3SS secretin) | 1.90895 | cellular response to oxidative stress [GO:0034599]; heme catabolic process [GO:0042167] |  | ferredoxin-NADP+ reductase activity [GO:0004324]; nucleotide binding [GO:0000166] |
| DAPPPG215_23735 | Periplasmic ligand-binding sensor protein | 1.80938 | protein secretion by the type III secretion system [GO:0030254] | cell outer membrane [GO:0009279]; type II protein secretion system complex [GO:0015627]; type III protein secretion system complex [GO:0030257] |  |
| DAPPPG215_14675 | Sulphite reductase | 1.77579 |  |  |  |
| DAPPPG215_16180 | Rrf2 family transcriptional regulator | 1.7636 |  |  | 4 iron, 4 sulfur cluster binding [GO:0051539]; heme binding [GO:0020037]; metal ion binding [GO:0046872]; oxidoreductase activity [GO:0016491] |
| DAPPPG215_03880 | Chaperone protein ClpB | 1.74692 |  | cytosol [GO:0005829] | DNA-binding transcription factor activity [GO:0003700] |
| DAPPPG215_18510 | RING-type E3 ubiquitin transferase (EC 2.3.2.27) | 1.66878 | cellular response to heat [GO:0034605]; protein refolding [GO:0042026] | cytoplasm [GO:0005737] | ATP binding [GO:0005524]; ATP hydrolysis activity [GO:0016887] |
| DAPPPG215_01835 | HTH-type transcriptional regulator BetI | 1.65924 | protein ubiquitination [GO:0016567] | cytoplasm [GO:0005737] | ubiquitin-protein transferase activity [GO:0004842] |
| DAPPPG215_01365 | Fructose-1,6-bisphosphate aldolase (FBP aldolase) (EC 4.1.2.13) | 1.65014 | glycine betaine biosynthetic process from choline [GO:0019285]; negative regulation of DNA-templated transcription [GO:0045892] |  | DNA-binding transcription factor activity [GO:0003700]; transcription cis-regulatory region binding [GO:0000976] |
| DAPPPG215_17645 | UvrABC system protein B (Protein UvrB) (Excinuclease ABC subunit B) | 1.63995 | glycolytic process [GO:0006096] |  | fructose-bisphosphate aldolase activity [GO:0004332]; zinc ion binding [GO:0008270] |
| DAPPPG215_21795 | Lon protease (EC 3.4.21.53) (ATP-dependent protease La) | 1.61569 | nucleotide-excision repair [GO:0006289]; SOS response [GO:0009432] | cytoplasm [GO:0005737]; excinuclease repair complex [GO:0009380] | ATP binding [GO:0005524]; ATP hydrolysis activity [GO:0016887]; DNA binding [GO:0003677]; excinuclease ABC activity [GO:0009381] |
| DAPPPG215_22485 | Cytoplasmic membrane family protein | 1.60087 | cellular response to heat [GO:0034605]; protein quality control for misfolded or incompletely synthesized proteins [GO:0006515] | cytoplasm [GO:0005737] | ATP binding [GO:0005524]; ATP hydrolysis activity [GO:0016887]; ATP-dependent peptidase activity [GO:0004176]; sequence-specific DNA binding [GO:0043565]; serine-type endopeptidase activity [GO:0004252] |
| DAPPPG215_29090 | Uncharacterized protein | 1.57907 |  | membrane [GO:0016020] |  |
| DAPPPG215_27255 | Major capsid protein | 1.56763 |  |  |  |
| DAPPPG215_22910 | Hydrolase, carbon-nitrogen family | 1.56573 |  |  |  |
| DAPPPG215_03035 | TonB system transport protein, putative | 1.56085 |  |  | hydrolase activity, acting on carbon-nitrogen (but not peptide) bonds, in linear amides [GO:0016811] |
| DAPPPG215_23525 | Type III chaperone ShcS2 | 1.55541 |  | cell outer membrane [GO:0009279] |  |
| DAPPPG215_10730 | Ankyrin domain protein | 1.55036 | protein secretion by the type III secretion system [GO:0030254] |  |  |
| DAPPPG215_22905 | TldD protein | 1.52968 |  |  |  |
| DAPPPG215_24425 | Curved DNA-binding protein | 1.51380 | proteolysis [GO:0006508] | cytosol [GO:0005829] | metallopeptidase activity [GO:0008237] |
| DAPPPG215_01370 | Urease accessory protein UreG | 1.50862 | chaperone cofactor-dependent protein refolding [GO:0051085]; protein refolding [GO:0042026] | cytoplasm [GO:0005737]; nucleoid [GO:0009295] | bent DNA binding [GO:0003681]; unfolded protein binding [GO:0051082] |
| DAPPPG215_08655 | Conserved domain protein | 1.50108 | urea catabolic process [GO:0043419] | cytoplasm [GO:0005737] | GTP binding [GO:0005525]; GTPase activity [GO:0003924]; nickel cation binding [GO:0016151] |
| DAPPPG215_14185 | Regulatory protein, putative | -1.50499 | DNA transposition [GO:0006313] |  | DNA binding [GO:0003677]; transposase activity [GO:0004803] |
| DAPPPG215_15900 | TonB protein | -1.51356 | protein transport [GO:0015031]; transmembrane transport [GO:0055085] | plasma membrane protein complex [GO:0098797] | energy transducer activity [GO:0031992] |
| DAPPPG215_01615 | Iron-sulfur cluster-binding protein | -1.52067 |  | plasma membrane [GO:0005886] | 4 iron, 4 sulfur cluster binding [GO:0051539]; metal ion binding [GO:0046872]; oxidoreductase activity [GO:0016491] |
| DAPPPG215_28265 | ABC transporter, permease protein | -1.53591 | transmembrane transport [GO:0055085] | plasma membrane [GO:0005886] |  |
| DAPPPG215_21635 | 5-methyltetrahydropteroyltriglutamate--homocysteine methyltransferase (EC 2.1.1.14) (Cobalamin-independent methionine synthase) (Methionine synthase, vitamin-B12 independent isozyme) | -1.53702 | methionine biosynthetic process [GO:0009086]; methylation [GO:0032259] |  | 5-methyltetrahydropteroyltriglutamate-homocysteine S-methyltransferase activity [GO:0003871]; zinc ion binding [GO:0008270] |
| DAPPPG215_00240 | Biopolymer transport protein ExbB | -1.54066 | protein import [GO:0017038] | plasma membrane [GO:0005886] | transmembrane transporter activity [GO:0022857] |
| DAPPPG215_12155 | Dipeptide ABC transporter, permease protein DppB, putative | -1.54072 |  | plasma membrane [GO:0005886] | dipeptide transmembrane transporter activity [GO:0071916] |
| DAPPPG215_10845 | succinate dehydrogenase (EC 1.3.5.1) | -1.5422 | respiratory electron transport chain [GO:0022904]; tricarboxylic acid cycle [GO:0006099] |  | 2 iron, 2 sulfur cluster binding [GO:0051537]; 3 iron, 4 sulfur cluster binding [GO:0051538]; 4 iron, 4 sulfur cluster binding [GO:0051539]; electron transfer activity [GO:0009055]; metal ion binding [GO:0046872]; oxidoreductase activity [GO:0016491] |
| DAPPPG215_10660 | Ferrous iron transport periplasmic protein EfeO | -1.57576 |  | membrane [GO:0016020]; periplasmic space [GO:0042597] |  |
| DAPPPG215_15315 | Yersiniabactin synthetase, thioesterase component | -1.57846 | lipid biosynthetic process [GO:0008610] |  |  |
| DAPPPG215_11940 | TonB-dependent siderophore receptor, putative | -1.58247 |  | cell outer membrane [GO:0009279] | siderophore uptake transmembrane transporter activity [GO:0015344]; signaling receptor activity [GO:0038023] |
| DAPPPG215_10655 | Deferrochelatase (EC 1.11.1.-) (Peroxidase EfeB) | -1.58257 | iron import into cell [GO:0033212] | cell envelope [GO:0030313]; cytosol [GO:0005829]; periplasmic space [GO:0042597] | ferrochelatase activity [GO:0004325]; heme binding [GO:0020037]; metal ion binding [GO:0046872]; peroxidase activity [GO:0004601] |
| DAPPPG215_16960 | Nitrate reductase | -1.58503 | cellular respiration [GO:0045333]; nitrate assimilation [GO:0042128] | membrane [GO:0016020]; oxidoreductase complex [GO:1990204] | 4 iron, 4 sulfur cluster binding [GO:0051539]; metal ion binding [GO:0046872]; molybdopterin cofactor binding [GO:0043546]; oxidoreductase activity [GO:0016491] |
| DAPPPG215_17785 | 2,4-diaminobutyrate 4-transaminase | -1.61209 | biosynthetic process [GO:0009058] |  | pyridoxal phosphate binding [GO:0030170]; transaminase activity [GO:0008483] |
| DAPPPG215_15895 | TonB system transport protein ExbB | -1.63141 | protein import [GO:0017038] | plasma membrane [GO:0005886] |  |
| DAPPPG215_16150 | Urease accessory protein UreF 1 | -1.64034 |  | cytoplasm [GO:0005737] | nickel cation binding [GO:0016151] |
| DAPPPG215_03675 | histidine kinase (EC 2.7.13.3) | -1.67369 |  | plasma membrane [GO:0005886] | phosphorelay sensor kinase activity [GO:0000155] |
| DAPPPG215_17730 | Pyoverdine sidechain peptide synthetase II, D-Asp-L-Thr component | -1.67449 |  |  | catalytic activity [GO:0003824]; phosphopantetheine binding [GO:0031177] |
| DAPPPG215_17775 | ABC transporter, periplasmic substrate-binding protein, putative | -1.69746 | metal ion transport [GO:0030001] |  | metal ion binding [GO:0046872] |
| DAPPPG215_17750 | Iron-regulated membrane protein, putative | -1.72928 |  | membrane [GO:0016020] |  |
| DAPPPG215_17790 | Pyoverdine chromophore synthetase | -1.75467 | lipid biosynthetic process [GO:0008610] |  | catalytic activity [GO:0003824]; phosphopantetheine binding [GO:0031177] |
| DAPPPG215_10650 | Conserved domain protein | -1.76710 |  | cell envelope [GO:0030313] |  |
| DAPPPG215_25935 | Monooxygenase, DszA family | -1.80072 |  |  | monooxygenase activity [GO:0004497]; oxidoreductase activity, acting on paired donors, with incorporation or reduction of molecular oxygen [GO:0016705] |
| DAPPPG215_21120 | Branched-chain amino acid ABC transporter, permease protein | -1.80082 | amino acid transport [GO:0006865] | plasma membrane [GO:0005886] | transmembrane transporter activity [GO:0022857] |
| DAPPPG215_12200 | Phytase domain protein | -1.85234 |  |  | 3-phytase activity [GO:0016158] |
| DAPPPG215_01200 | Lipase family protein | -1.86718 | lipid metabolic process [GO:0006629] |  |  |
| DAPPPG215_15430 | Peptide ABC transporter, permease protein | -1.89851 |  | plasma membrane [GO:0005886] | dipeptide transmembrane transporter activity [GO:0071916] |
| DAPPPG215_17760 | High-affinity zinc uptake system protein ZnuA | -1.90998 | cell adhesion [GO:0007155]; metal ion transport [GO:0030001] |  | metal ion binding [GO:0046872] |
| DAPPPG215_16600 | Type III effector HopI1 | -1.91450 | effector-mediated suppression of host salicylic acid-mediated innate immune signaling [GO:0140502] | host cell chloroplast [GO:0033652] |  |
| DAPPPG215_13445 | Branched-chain amino acid ABC transporter, permease protein | -1.93910 | amino acid transport [GO:0006865] | plasma membrane [GO:0005886] | transmembrane transporter activity [GO:0022857] |
| DAPPPG215_15700 | ABC transporter, permease protein | -1.99463 | transmembrane transport [GO:0055085] | plasma membrane [GO:0005886] |  |
| DAPPPG215_16155 | Urease subunit gamma/beta (EC 3.5.1.5) (Urea amidohydrolase subunit gamma/beta) | -1.99839 | urea catabolic process [GO:0043419] | urease complex [GO:0035550] | nickel cation binding [GO:0016151]; urease activity [GO:0009039] |
| DAPPPG215_13805 | Aminotransferase (EC 2.6.1.-) | -2.04301 | amino acid metabolic process [GO:0006520]; biosynthetic process [GO:0009058] |  | pyridoxal phosphate binding [GO:0030170]; transaminase activity [GO:0008483] |
| DAPPPG215_17665 | Efflux transporter, RND family, MFP subunit | -2.07010 | xenobiotic detoxification by transmembrane export across the plasma membrane [GO:1990961] | cell envelope [GO:0030313]; efflux pump complex [GO:1990281]; extrinsic component of membrane [GO:0019898]; macrolide transmembrane transporter complex [GO:1990195] | efflux transmembrane transporter activity [GO:0015562] |
| DAPPPG215_00250 | Protein TonB | -2.07045 | protein transport [GO:0015031]; siderophore transport [GO:0015891]; transmembrane transport [GO:0055085] | outer membrane-bounded periplasmic space [GO:0030288]; plasma membrane protein complex [GO:0098797] | energy transducer activity [GO:0031992] |
| DAPPPG215_17800 | RNA polymerase sigma-70 family protein | -2.13143 | DNA-templated transcription initiation [GO:0006352]; positive regulation of DNA-templated transcription [GO:0045893] | protein-DNA complex [GO:0032993] | DNA-binding transcription activator activity [GO:0001216]; sigma factor activity [GO:0016987]; transcription cis-regulatory region binding [GO:0000976] |
| DAPPPG215_00635 | TonB protein, putative | -2.13499 | protein transport [GO:0015031]; transmembrane transport [GO:0055085] | plasma membrane protein complex [GO:0098797] | energy transducer activity [GO:0031992] |
| DAPPPG215_00040 | FAD/NAD(P)-binding domain-containing protein | -2.29689 | sulfide oxidation, using sulfide:quinone oxidoreductase [GO:0070221] |  | FAD binding [GO:0071949]; sulfide:quinone oxidoreductase activity [GO:0070224] |
| DAPPPG215_06040 | Regulatory protein, putative | -2.48887 | iron ion transport [GO:0006826]; regulation of DNA-binding transcription factor activity [GO:0051090] |  | sigma factor antagonist activity [GO:0016989] |
| DAPPPG215_15435 | Peptide ABC transporter, permease protein | -2.49354 | transmembrane transport [GO:0055085] | plasma membrane [GO:0005886] |  |
| DAPPPG215_00035 | Metallo-beta-lactamase superfamily protein | -2.56067 | glutathione metabolic process [GO:0006749]; hydrogen sulfide metabolic process [GO:0070813] |  | hydrolase activity [GO:0016787]; metal ion binding [GO:0046872]; sulfur dioxygenase activity [GO:0050313] |
| DAPPPG215_27625 | Uncharacterized protein | -Inf |  | membrane [GO:0016020] |  |
| DAPPPG215_24010 | Rrf2 family protein | -Inf |  |  |  |

**Table S4.** List of differentially expressed genes (DEGs) in *Pseudomonas syringae* pv. tomato strain DAPP-PG 215 exposed to Argirium-SUNCs for 30 minutes (*T_30_*) with their UniProt ID, BLAST annotation and log_2_-Fold Change

| **Locus tag** | **Annotation** | **log_2_-Fold Change** | **Gene Ontology**  **(biological process)** | **Gene Ontology**  **(cellular component)** | **Gene Ontology**  **(molecular function)** |
| --- | --- | --- | --- | --- | --- |
| DAPPPG215_23325 | DUF2188 domain-containing protein | Inf |  |  |  |
| DAPPPG215_19760 | Fe-S oxidoreductase | Inf |  |  |  |
| DAPPPG215_26360 | Cadmium-translocating P-type ATPase | 3.91872 |  | plasma membrane [GO:0005886] | ATP binding [GO:0005524]; ATP hydrolysis activity [GO:0016887]; ATPase-coupled monoatomic cation transmembrane transporter activity [GO:0019829]; cadmium ion transmembrane transporter activity [GO:0015086]; metal ion binding [GO:0046872]; P-type ion transporter activity [GO:0015662] |
| DAPPPG215_01380 | Transcriptional regulator, ArsR family | 3.26788 |  |  | DNA-binding transcription factor activity [GO:0003700] |
| DAPPPG215_01280 | Efflux RND transporter permease subunit | 3.17910 |  | plasma membrane [GO:0005886] | monoatomic cation transmembrane transporter activity [GO:0008324]; xenobiotic transmembrane transporter activity [GO:0042910] |
| DAPPPG215_10940 | C4-dicarboxylate transporter/malic acid transport protein | 2.61760 |  | plasma membrane [GO:0005886] | sulfite transmembrane transporter activity [GO:0000319] |
| DAPPPG215_06925 | Type 3 secretion system secretin (T3SS secretin) | 2.54669 | protein secretion by the type III secretion system [GO:0030254] | cell outer membrane [GO:0009279]; type II protein secretion system complex [GO:0015627] |  |
| DAPPPG215_07725 | Tellurite resistance TerB family protein | 2.37675 |  |  |  |
| DAPPPG215_01865 | Membrane protein, putative | 2.31053 |  | membrane [GO:0016020] |  |
| DAPPPG215_03455 | Bcr/CflA family efflux transporter | 2.23484 | xenobiotic detoxification by transmembrane export across the plasma membrane [GO:1990961] | plasma membrane [GO:0005886] | sodium:proton antiporter activity [GO:0015385]; xenobiotic transmembrane transporter activity [GO:0042910] |
| DAPPPG215_21935 | Lipoprotein, putative | 2.22974 |  |  |  |
| DAPPPG215_12990 | Major facilitator family transporter | 2.2031 |  | plasma membrane [GO:0005886] | transmembrane transporter activity [GO:0022857] |
| DAPPPG215_19955 | Protease HtpX (EC 3.4.24.-) (Heat shock protein HtpX) | 2.12885 | proteolysis [GO:0006508] | plasma membrane [GO:0005886] | metalloendopeptidase activity [GO:0004222]; zinc ion binding [GO:0008270] |
| DAPPPG215_01370 | Urease accessory protein UreG | 2.11077 | urea catabolic process [GO:0043419] | cytoplasm [GO:0005737] | GTP binding [GO:0005525]; GTPase activity [GO:0003924]; nickel cation binding [GO:0016151] |
| DAPPPG215_01375 | Arsenical pump membrane protein | 2.11052 | response to arsenic-containing substance [GO:0046685] | plasma membrane [GO:0005886] | antimonite secondary active transmembrane transporter activity [GO:0042960]; arsenite secondary active transmembrane transporter activity [GO:0008490] |
| DAPPPG215_26355 | Formimidoylglutamase, putative | 2.10557 |  |  |  |
| DAPPPG215_07695 | Conserved domain protein | 2.01037 |  |  |  |
| DAPPPG215_25700 | ATP-dependent protease subunit HslV (EC 3.4.25.2) | 1.99673 | proteolysis involved in protein catabolic process [GO:0051603] | HslUV protease complex [GO:0009376]; proteasome core complex [GO:0005839] | metal ion binding [GO:0046872]; threonine-type endopeptidase activity [GO:0004298] |
| DAPPPG215_17900 | DUF1652 domain-containing protein | 1.97512 |  |  |  |
| DAPPPG215_16180 | Rrf2 family transcriptional regulator | 1.97253 |  | cytosol [GO:0005829] | DNA-binding transcription factor activity [GO:0003700] |
| DAPPPG215_20855 | Lipoprotein | 1.92274 |  |  |  |
| DAPPPG215_12480 | Lipoprotein, putative | 1.92135 | response to desiccation [GO:0009269] |  |  |
| DAPPPG215_08170 | ferredoxin--NADP(+) reductase (EC 1.18.1.2) | 1.91252 | cellular response to oxidative stress [GO:0034599]; heme catabolic process [GO:0042167] |  | ferredoxin-NADP+ reductase activity [GO:0004324]; nucleotide binding [GO:0000166] |
| DAPPPG215_18645 | DUF3509 domain-containing protein | 1.87265 |  |  |  |
| DAPPPG215_00915 | Conserved domain protein | 1.87155 |  |  |  |
| DAPPPG215_23735 | Periplasmic ligand-binding sensor protein | 1.86678 |  |  |  |
| DAPPPG215_24425 | Curved DNA-binding protein | 1.85600 | chaperone cofactor-dependent protein refolding [GO:0051085]; protein refolding [GO:0042026] | cytoplasm [GO:0005737]; nucleoid [GO:0009295] | bent DNA binding [GO:0003681]; unfolded protein binding [GO:0051082] |
| DAPPPG215_25210 | L-threonate dehydrogenase (EC 1.1.1.411) | 1.84902 | organic acid catabolic process [GO:0016054] |  | NAD binding [GO:0051287]; NADP binding [GO:0050661]; oxidoreductase activity, acting on the CH-OH group of donors, NAD or NADP as acceptor [GO:0016616] |
| DAPPPG215_28310 | Type III helper protein HopAK1 | 1.84688 | polysaccharide catabolic process [GO:0000272] | extracellular region [GO:0005576] | pectate lyase activity [GO:0030570] |
| DAPPPG215_03405 | Oxidoreductase, short chain dehydrogenase/reductase family | 1.82793 |  |  | oxidoreductase activity, acting on the CH-OH group of donors, NAD or NADP as acceptor [GO:0016616] |
| DAPPPG215_07365 | Glycine zipper 2TM domain-containing protein | 1.81349 |  | outer membrane [GO:0019867] |  |
| DAPPPG215_01365 | Fructose-1,6-bisphosphate aldolase (FBP aldolase) (EC 4.1.2.13) | 1.79108 | glycolytic process [GO:0006096] |  | fructose-bisphosphate aldolase activity [GO:0004332]; zinc ion binding [GO:0008270] |
| DAPPPG215_19315 | SH3b domain-containing protein | 1.76806 |  | membrane [GO:0016020] |  |
| DAPPPG215_27315 | Phosphatidate cytidylyltransferase (EC 2.7.7.41) | 1.75474 | CDP-diacylglycerol biosynthetic process [GO:0016024] | plasma membrane [GO:0005886] | phosphatidate cytidylyltransferase activity [GO:0004605] |
| DAPPPG215_14675 | Sulphite reductase | 1.74851 |  |  | 4 iron, 4 sulfur cluster binding [GO:0051539]; heme binding [GO:0020037]; metal ion binding [GO:0046872]; oxidoreductase activity [GO:0016491] |
| DAPPPG215_17700 | Pyoverdine ABC transporter, ATP-binding/permease protein | 1.73538 | peptide transport [GO:0015833] | plasma membrane [GO:0005886] | ABC-type transporter activity [GO:0140359]; ATP binding [GO:0005524]; ATP hydrolysis activity [GO:0016887]; ATPase-coupled lipid transmembrane transporter activity [GO:0034040]; peptide transmembrane transporter activity [GO:1904680] |
| DAPPPG215_05855 | OmpA family protein | 1.70921 |  | cell outer membrane [GO:0009279] |  |
| DAPPPG215_01500 | DUF1795 domain-containing protein | 1.70914 |  |  |  |
| DAPPPG215_22485 | Cytoplasmic membrane family protein | 1.67995 |  | membrane [GO:0016020] |  |
| DAPPPG215_18680 | Serine aminopeptidase S33 domain-containing protein | 1.66486 |  |  | carboxylic ester hydrolase activity [GO:0052689] |
| DAPPPG215_01885 | Acyltransferase family protein | 1.66358 |  | membrane [GO:0016020] | acyltransferase activity [GO:0016746] |
| DAPPPG215_17240 | Lipoprotein, putative | 1.65456 |  |  |  |
| DAPPPG215_26005 | Dioxygenase, TauD/TfdA family | 1.63185 | sulfur compound metabolic process [GO:0006790] | cytoplasm [GO:0005737] | taurine dioxygenase activity [GO:0000908] |
| DAPPPG215_21870 | RNA polymerase sigma factor | 1.62302 | DNA-templated transcription initiation [GO:0006352] |  | DNA binding [GO:0003677]; sigma factor activity [GO:0016987] |
| DAPPPG215_10195 | Uncharacterized protein | 1.61111 |  |  |  |
| DAPPPG215_28165 | DUF3617 domain-containing protein | 1.60501 |  |  |  |
| DAPPPG215_01275 | RarD protein | 1.59954 |  | plasma membrane [GO:0005886] |  |
| DAPPPG215_00840 | DUF2388 domain-containing protein | 1.5644 |  |  |  |
| DAPPPG215_24795 | Cytosine/purine/uracil/thiamine/allantoin permease family protein | 1.56206 |  | plasma membrane [GO:0005886] | cytosine transmembrane transporter activity [GO:0015209] |
| DAPPPG215_19485 | histidine kinase (EC 2.7.13.3) | 1.55895 |  |  | phosphorelay sensor kinase activity [GO:0000155] |
| DAPPPG215_16125 | Branched-chain amino acid ABC transporter, permease protein | 1.55588 | amino acid transport [GO:0006865] | plasma membrane [GO:0005886] | transmembrane transporter activity [GO:0022857] |
| DAPPPG215_18640 | Ankyrin domain protein | 1.54933 |  | cytoplasm [GO:0005737] |  |
| DAPPPG215_20850 | Glucose/Sorbosone dehydrogenase domain-containing protein | 1.54797 |  |  |  |
| DAPPPG215_28635 | Bacterial luciferase family protein | 1.54691 |  | cytosol [GO:0005829] | oxidoreductase activity, acting on paired donors, with incorporation or reduction of molecular oxygen [GO:0016705] |
| DAPPPG215_21865 | Sigma factor algU negative regulatory protein MucA | 1.52882 |  | plasma membrane [GO:0005886] | sigma factor antagonist activity [GO:0016989] |
| DAPPPG215_01400 | DUF2790 domain-containing protein | 1.52629 |  |  |  |
| DAPPPG215_22910 | Hydrolase, carbon-nitrogen family | 1.51633 |  |  | hydrolase activity, acting on carbon-nitrogen (but not peptide) bonds, in linear amides [GO:0016811] |
| DAPPPG215_27150 | C4-type zinc finger protein, DksA/TraR family | -1.55090 | positive regulation of secondary metabolite biosynthetic process [GO:1900378] |  | zinc ion binding [GO:0008270] |
| DAPPPG215_06035 | Iron(III) dicitrate transport protein fecA | -1.66831 |  | cell outer membrane [GO:0009279] | siderophore-iron transmembrane transporter activity [GO:0015343]; signaling receptor activity [GO:0038023] |
| DAPPPG215_10650 | Conserved domain protein | -1.82872 |  | cell envelope [GO:0030313] |  |
| DAPPPG215_00040 | FAD/NAD(P)-binding domain-containing protein | -2.22718 | sulfide oxidation, using sulfide:quinone oxidoreductase [GO:0070221] |  | FAD binding [GO:0071949]; sulfide:quinone oxidoreductase activity [GO:0070224] |
| DAPPPG215_18465 | Uncharacterized protein | -2.69696 |  |  |  |
| DAPPPG215_00035 | AAA+ ATPase domain-containing protein | -2.88894 |  |  | ATP binding [GO:0005524]; ATP hydrolysis activity [GO:0016887] |
| DAPPPG215_02515 | IS, phage, Tn3B Transposon-related function | -3.00682 |  |  |  |
| DAPPPG215_13110 | Metallo-beta-lactamase superfamily protein | -3.06445 | glutathione metabolic process [GO:0006749]; hydrogen sulfide metabolic process [GO:0070813] |  | hydrolase activity [GO:0016787]; metal ion binding [GO:0046872]; sulfur dioxygenase activity [GO:0050313] |
| DAPPPG215_18335 | Transposase | -Inf | DNA integration [GO:0015074] |  | nucleic acid binding [GO:0003676] |

**Table S5.** Gene-list enrichment analysis results based on KEGG pathway and GO term functional categories in the Argirium-SUNCs-treated group following a 10-minutes exposure.

| **Term** | **Input genes** | **Total genes** | ***P*-Value** | **Corrected *P*-Value** |
| --- | --- | --- | --- | --- |
| Quorum sensing | DAPPPG215_12155 DAPPPG215_13445 DAPPPG215_15430  DAPPPG215_15435  DAPPPG215_15700 | 95 | 0.011 | 0.440 |
| 2-Oxocarboxylic acid metabolism | DAPPPG215_13805  DAPPPG215_17785 | 25 | 0.0509 | 0.463 |
| Arginine biosynthesis | DAPPPG215_13805  DAPPPG215_06040 | 25 | 0.0509 | 0.463 |
| Novobiocin biosynthesis | DAPPPG215_13805 | 5 | 0.0785 | 0.463 |
| Biosynthesis of amino acids | DAPPPG215_01365  DAPPPG215_13805 DAPPPG215_17785  DAPPPG215_21635 | 126 | 0.0984 | 0.463 |
| Sulfur metabolism | DAPPPG215_00040 DAPPPG215_14675 | 39 | 0.106 | 0.463 |
| Cysteine and methionine metabolism | DAPPPG215_13805  DAPPPG215_21635 | 41 | 0.114 | 0.463 |
| Atrazine degradation | DAPPPG215_06040 | 8 | 0.115 | 0.463 |
| Microbial metabolism in diverse environments | DAPPPG215_10845 DAPPPG215_01365  DAPPPG215_14675 DAPPPG215_16960 DAPPPG215_06040 DAPPPG215_17785 | 242 | 0.118 | 0.463 |
| Nucleotide excision repair | DAPPPG215_17645 | 9 | 0.127 | 0.463 |
| Pentose and glucuronate interconversions | DAPPPG215_13965 | 9 | 0.127 | 0.463 |
| Selenocompound metabolism | DAPPPG215_21635 | 10 | 0.139 | 0.464 |
| Inositol phosphate metabolism | DAPPPG215_12200 | 15 | 0.196 | 0.48 |
| Ascorbate and aldarate metabolism | DAPPPG215_13965 | 15 | 0.196 | 0.48 |
| Tyrosine metabolism | DAPPPG215_13805 | 16 | 0.207 | 0.48 |
| Phenylalanine metabolism | DAPPPG215_13805 | 18 | 0.228 | 0.48 |
| Nitrogen metabolism | DAPPPG215_16960 | 20 | 0.249 | 0.48 |
| Fructose and mannose metabolism | DAPPPG215_01365 | 21 | 0.259 | 0.48 |
| Histidine metabolism | DAPPPG215_26355 | 22 | 0.269 | 0.48 |
| Glycerophospholipid metabolism | DAPPPG215_27315 | 22 | 0.269 | 0.48 |
| Methane metabolism | DAPPPG215_01365 | 23 | 0.279 | 0.48 |
| Citrate cycle (TCA cycle) | DAPPPG215_10845 | 25 | 0.299 | 0.48 |
| Phenylalanine, tyrosine and tr... | DAPPPG215_13805 | 28 | 0.327 | 0.48 |
| Amino sugar and nucleotide sugar metabolism | DAPPPG215_13965 | 28 | 0.327 | 0.48 |
| Butanoate metabolism | DAPPPG215_10845 | 29 | 0.336 | 0.48 |
| Glycolysis / Gluconeogenesis | DAPPPG215_01365 | 30 | 0.345 | 0.48 |
| Arginine and proline metabolism | DAPPPG215_13805 | 31 | 0.354 | 0.48 |
| Pentose phosphate pathway | DAPPPG215_01365 | 31 | 0.354 | 0.48 |
| Alanine, aspartate and glutamate metabolism | DAPPPG215_13805 | 31 | 0.354 | 0.48 |
| Metabolic pathways | DAPPPG215_12200  DAPPPG215_01365 DAPPPG215_08170 DAPPPG215_10845  DAPPPG215_13805  DAPPPG215_13965  DAPPPG215_14675 DAPPPG215_06040 DAPPPG215_16960 DAPPPG215_17785  DAPPPG215_21635 DAPPPG215_27315  DAPPPG215_26355 | 837 | 0.36 | 0.48 |
| Biosynthesis of secondary metabolites | DAPPPG215_01365 DAPPPG215_10845  DAPPPG215_13805 DAPPPG215_21635 DAPPPG215_27315 | 301 | 0.399 | 0.514 |
| Oxidative phosphorylation | DAPPPG215_10845 | 41 | 0.437 | 0.532 |
| Carbon metabolism | DAPPPG215_01365 DAPPPG215_10845 | 108 | 0.44 | 0.532 |
| Glyoxylate and dicarboxylate metabolism | DAPPPG215_26355 | 43 | 0.452 | 0.532 |
| Glycine, serine and threonine metabolism | DAPPPG215_17785 | 47 | 0.481 | 0.55 |
| Bacterial secretion system | DAPPPG215_06925 | 57 | 0.548 | 0.609 |
| Biosynthesis of antibiotics | DAPPPG215_01365 DAPPPG215_10845 DAPPPG215_13805 | 225 | 0.601 | 0.65 |
| Purine metabolism | DAPPPG215_06040 | 71 | 0.627 | 0.65 |
| ABC transporters | DAPPPG215_13445 DAPPPG215_17760 DAPPPG215_21120 | 236 | 0.634 | 0.65 |
| Two-component system | DAPPPG215_06040 | 188 | 0.927 | 0.927 |

**Table S6.** Gene-list enrichment analysis results based on KEGG pathway and GO term functional categories in the Argirium-SUNCs-treated group following a 30-minutes exposure.

| **Term** | **Input genes** | **Total genes** | ***P*-Value** | **Corrected *P*-Value** |
| --- | --- | --- | --- | --- |
| Metabolic pathways | DAPPPG215_01365  DAPPPG215_08170  DAPPPG215_14675  DAPPPG215_26355  DAPPPG215_27315 | 837 | 0.941 | 0.941 |
| Biosynthesis of secondary metabolism | DAPPPG215_01365  DAPPPG215_27315 | 301 | 0.875 | 0.921 |
| Microbial metabolism in diverse environments | DAPPPG215_01365  DAPPPG215_14675 | 242 | 0.783 | 0.870 |
| Biosynthesis of amino acids | DAPPPG215_01365 | 126 | 0.777 | 0.870 |
| ABC transporters | DAPPPG215_16125  DAPPPG215_17700 | 236 | 0.771 | 0.870 |
| Biosynthesis of antibiotics | DAPPPG215_03405 DAPPPG215_01365 | 225 | 0.747 | 0.870 |
| Carbon metabolism | DAPPPG215_01365 | 108 | 0.723 | 0.870 |
| Bacterial secretion system | DAPPPG215_06925 | 57 | 0.494 | 0.760 |
| Glyoxylate and dicarboxylate metabolism | DAPPPG215_26355 | 43 | 0.403 | 0.672 |
| Fatty acid metabolism | DAPPPG215_03405 | 35 | 0.344 | 0.626 |
| Pentose phosphate pathway | DAPPPG215_01365 | 31 | 0.313 | 0.625 |
| Glycolysis / Gluconeogenesis | DAPPPG215_01365 | 30 | 0.304 | 0.625 |
| Fatty acid biosynthesis | DAPPPG215_03405 | 24 | 0.254 | 0.625 |
| Methane metabolism | DAPPPG215_01365 | 23 | 0.245 | 0.625 |
| Glycerophospholipid metabolism | DAPPPG215_27315 | 22 | 0.236 | 0.625 |
| Histidine metabolism | DAPPPG215_26355 | 22 | 0.236 | 0.625 |
| Fructose and mannose metabolism | DAPPPG215_01365 | 21 | 0.227 | 0.625 |
| Biotin metabolism | DAPPPG215_03405 | 19 | 0.209 | 0.625 |
| Taurine and hypotaurine metabolism | DAPPPG215_26005 | 5 | 0.0677 | 0.625 |
| Sulfur metabolism | DAPPPG215_00040 DAPPPG215_14675  DAPPPG215_26005 | 39 | 0.0124 | 0.249 |
